# Anti-inflammation is an important way that Qingre-Huazhuo-Jiangsuan recipe treats acute gouty arthritis

**DOI:** 10.3389/fphar.2023.1268641

**Published:** 2023-10-10

**Authors:** Yazhuo Wang, Yang Xu, Jingrui Tan, Jiaxue Ye, Weizhen Cui, Jie Hou, Peiyu Liu, Jianwei Li, Shiyuan Wang, Qingyang Zhao

**Affiliations:** ^1^ Institute of Traditional Chinese Medicine, Shandong University of Traditional Chinese Medicine, Jinan, China; ^2^ Institute of Nursing, Shandong University of Traditional Chinese Medicine, Jinan, China

**Keywords:** QHJR, AGA, apoptosis, autophagy, inflammation, gene

## Abstract

**Background:** Acute gouty arthritis (AGA) significantly impairs patients’ quality of life. Currently, existing therapeutic agents exhibit definite efficacy but also lead to serious adverse reactions. Therefore, it is essential to develop highly efficient therapeutic agents with minimal adverse reactions, especially within traditional Chinese medicine (TCM). Additionally, food polyphenols have shown potential in treating various inflammatory diseases. The Qingre-Huazhuo-Jiangsuan-Recipe (QHJR), a modification of Si-Miao-San (SMS), has emerged as a TCM remedy for AGA with no reported side effects. Recent research has also highlighted a strong genetic link to gout.

**Methods:** The TCM System Pharmacology (TCMSP) database was used to collect the main chemical components of QHJR and AGA-related targets for predicting the metabolites in QHJR. HPLC-Q-Orbitrap-MS was employed to identify the ingredients of QHJR. The collected metabolites were then used to construct a Drugs-Targets Network in Cytoscape software, ranked based on their “Degree” of significance. Differentially expressed genes (DEGs) were screened in the Gene Expression Omnibus (GEO) database using GEO2R online analysis. Subsequently, Gene Ontology (GO) and Kyoto Encyclopedia of Genes and Genomes (KEGG) enrichment analyses were performed. The DEGs were utilized to construct a Protein-Protein Interaction (PPI) Network via the STRING database. *In vivo* experimental validation was conducted using colchicine, QHJR, rapamycin (RAPA), and 3-methyladenine (3-MA) as controls to observe QHJR’s efficacy in AGA. Synovial tissues from rats were collected, and qRT-PCR and Western blot assays were employed to investigate Ampk-related factors (Ampk, mTOR, ULK1), autophagy-related factors (Atg5, Atg7, LC3, p62), and inflammatory-related factors (NLRP3). ELISA assays were performed to measure inflammatory-related factor levels (IL-6, IL-1β, TNF-α), and H&E staining was used to examine tissue histology.

**Results:** Network analysis screened out a total of 94 metabolites in QHJR for AGA. HPLC-Q-Orbitrap-MS analysis identified 27 of these metabolites. Notably, five metabolites (Neochlorogenic acid, Caffeic acid, Berberine, Isoliquiritigenin, Formononetin) were not associated with any individual herbal component of QHJR in TCMSP database, while six metabolites (quercetin, luteolin, formononetin, naringenin, taxifolin, diosgenin) overlapped with the predicted results from the previous network analysis. Further network analysis highlighted key components, such as Caffeic acid, cis-resveratrol, Apigenin, and Isoliquiritigenin. Other studies have found that their treatment of AGA is achieved through reducing inflammation, consistent with this study, laying the foundation for the mechanism study of QHJR against AGA. PPI analysis identified TNF, IL-6, and IL-1β as hub genes. GO and KEGG analyses indicated that anti-inflammation was a key mechanism in AGA treatment. All methods demonstrated that inflammatory expression increased in the Model group but was reversed by QHJR. Additionally, autophagy-related expression increased following QHJR treatment. The study suggested that AMPKα and p-AMPKα1 proteins were insensitive to 3 MA and RAPA, implying that AMPK may not activate autophagy directly but through ULK1 and mTOR.

**Conclusion:** In conclusion, this study confirms the effectiveness of QHJR, a modified formulation of SMS (a classic traditional Chinese medicine prescription for treating gout), against AGA. QHJR, as a TCM formula, offers advantages such as minimal safety concerns and potential long-term use. The study suggests that the mechanism by which QHJR treats AGA may involve the activation of the AMPK/mTOR/ULK1 pathway, thereby regulating autophagy levels, reducing inflammation, and alleviating AGA. These findings provide new therapeutic approaches and ideas for the clinical treatment of AGA.

## 1 Introduction

Acute gouty arthritis (AGA) represents a major and increasingly serious public health issue, whose incidence is increasing year by year and seriously impacting people’s health and lives (Zhu et al., 2011; Khanna et al., 2015; Terkeltaub, 2017). AGA is a common inflammatory disease, mainly manifested by a potent inflammatory response resulting from monosodium urate (MSU) crystal deposition in tissues including joints (Fischer et al., 2018; Gu et al., 2021). MSU crystals accumulate in periarticular tissues, which can stimulate joint synovium and produce pathological reactions including leucocyte exudate, synovial vasodilation, and enhanced permeability (Lyu et al., 2021). The existing anti-inflammatory drugs adopted for treating and preventing gouty arthritis (GA) have been limited (Gu et al., 2021). However, just 1/2 of AGA cases can respond to the existing therapeutics (Yuan et al., 2019; Gu et al., 2021). Therefore, it is essential to investigate the efficacy of Traditional Chinese Medicine (TCM).

Traditional Chinese Medicine (TCM) has been used to treat gouty arthritis (GA) for centuries. The most widely used TCM treatments for GA are acupuncture and the Chinese herbal medicine Si-Miao-San (Chi et al., 2020; Pu et al., 2021). Acupuncture is based on the theory of meridians, while SMS is a combination of four herbs that have been used to treat GA for over 1,000 years (Chi et al., 2020; Pu et al., 2021), SMS can significantly improve the symptoms of acute arthritis in gout patients, and its curative effect is not lower than colchicine, with small side effects (Chi et al., 2020; Pu et al., 2021). SMS is effective in the treatment of GA through anti-inflammation and lowering urate (Chi et al., 2020; Pu et al., 2021). Acupuncture treatment for GA involves the ing spleen, to the unifying kidney, dissipating dampness, resolving blood stasis, clearing away heat, and removing toxins (Chi et al., 2020; Pu et al., 2021). Qingre-Huazhuo-Jiangsuan Recipe (QHJR), one of the TCM prescriptions, is prepared based on the classic TCM prescription Si-Miao-San that can be empirically adopted for treating GA during the clinical practice of TCM (Fan et al., 2010). In traditional Chinese medicine, the pathological mechanism of GA is primarily believed to involve “damp-heat descending, leading to obstructing the meridians and the sluggish circulation of qi and blood.” However, AGA has a sudden onset, and its pathological mechanism is mainly characterized by “internal accumulation of damp-heat turbidity, which flows and affects the viscera and joints.” Therefore, based on SMS (a previous treatment method), modifications were made by retaining and increasing the dosage of Coix lacryma-jobi var. ma-yuen (Rom.Caill.) Stapf (Poaceae; Coicis semen) and Atractylodes Lancea (Thunb.) DC (Asteraceae; Atractylodis rhizoma), and incorporating other heat-clearing, detoxifying, and turbidity-resolving herbs to consolidate the treatment. QHJR is composed of 11 botanical drugs, including Reynoutria japonica Houtt (Polygonaceae; Polygoni cuspidati rhizoma et radix), Spatholobus suberectus Dunn (Fabaceae; Spatholobi caulis), Dioscorea septemloba Thunb (Dioscoreaceae; Dioscoreae spongiosae rhizoma), Atractylodes Lancea (Thunb.) DC (Asteraceae; Atractylodis rhizoma), Plantago depressa Willd (Plantaginaceae; Plantaginis semen), Clematis chinensis Osbeck (Ranunculaceae; Clematidis radix et rhizoma), Smilax glabra Roxb (Smilacaceae; Smilacis glabrae rhizoma), Poria cocos (Schw.) Wolf (Polyporaceae; Poria), Curcuma longa L (Zingiberaceae; Curcumae longae rhizoma), Perilla frutescens L.) Britton (Lamiaceae; Perillae folium) and Coix lacryma-jobi var. ma-yuen (Rom.Caill.) Stapf (Poaceae; Coicis semen). Please refer to Supplementary Table S1 for details. The drugs of first choice for acute gouty arthritis are nonsteroidal anti-inflammatory drugs (NSAID), corticosteroids, and colchicine (Engel et al., 2017). Colchicine is a well-established and affordable anti-inflammatory agent with a long history of use for treating various diseases, primarily rheumatic and cardiac conditions, as well as gout attacks (Thompson and Nidorf, 2018; El Hasbani et al., 2021). However, it is important to note that it has potential side effects, such as liver failure (Abbott et al., 2017), diarrhea (Tardif et al., 2019), and vomiting (Angelidis et al., 2018). Colchicine has been shown to have several mechanisms of action in the treatment of gout, including inhibiting the activation of the NLRP3 inflammasome, blocking the release of IL-1β (Cronstein and Terkeltaub, 2006; Nuki, 2008; Dalbeth et al., 2014). Its primary mode of treatment is anti-inflammatory, which is consistent with this study (Thompson and Nidorf, 2018). In this study, QHJR was compared to colchicine as a control group to investigate whether it could reduce inflammation consistently or not and to explore the mechanism of QHJR on AGA.

The molecules identified in gout are involved in a multitude of critical processes, including the degradation of the extracellular matrix in cartilage cells, transduction of inflammatory signals, and regulation of fibroblast-like synoviocyte invasion pathways (Liu et al., 2014; Chew et al., 2018; Shu et al., 2022). In addition, miRNAs and lncRNAs have been shown to play indispensable roles in the initiation of inflammation in acute gout as constituents of MSU-induced inflammatory pathways (Papanagnou et al., 2016; Xu B. Y. et al., 2020; Galozzi et al., 2021; Shu et al., 2022). The results underscore the potential of these molecules as targets for the prediction and management of AGA, thereby warranting further study and exploration.

Autophagy represents the basic intracellular decomposition event, and has different effects on immunity, thereby maintaining cell homeostasis while also determining their fate (Hao and Liu, 2021). Autophagy is a finely regulated cellular program in which multiple pathways play vital roles. During the last decade, human genetic research suggests that autophagy is tightly associated with autoimmune/inflammatory disorders and cancers (Lyu et al., 2021). Recent research shows that autophagy is related to arthritis progression. For example, based on Yun Yu et al. (Yu S. et al., 2020), autophagy expression has effects on arthritis. According to Piras et al. (Piras et al., 2017; Yang et al., 2021), autophagy suppression decreased bone erosion severity and osteoclast (OC) number, suggesting that the critical function of autophagy is protecting bone tissues. Studies have shown that autophagy plays a key role in the pathogenesis of GA (Han et al., 2021). MSU crystals, which are associated with gouty arthritis, have been shown to induce autophagy in macrophages and synovial tissues from patients with gouty arthritis (Xiao et al., 2023; Yuan et al., 2023). Autophagy-lysosomal pathway (ALP) perturbations have been observed in synovial macrophages from patients with gouty arthritis (Chen et al., 2023). Autophagy is involved in decreasing inflammation. It has been suggested that autophagy induced by *P*P121 can alleviate MSU crystal-induced acute gouty arthritis via inhibition of the NLRP3 inflammasome (Yuan et al., 2023). Autophagy inhibits inflammatory signaling complexes (Deretic and 2021). Autophagy may directly regulate inflammation by removing or down-regulating pro-inflammatory cytokines and degrading inflammasome components (Pang et al., 2022). Modulation of autophagy, which plays a critical role in inflammation by influencing the development, homeostasis, and survival of inflammatory cells, might lead to therapeutic interventions for diseases associated with inflammation (Qian et al., 2017). The aforementioned literature research highlights the critical role of autophagy in AGA. To validate the role of autophagy in inflammation, this study employed rapamycin (RAPA, an autophagy inducer) and 3-methyladenine (3-MA, an autophagy inhibitor) as control groups to observe changes in autophagy levels and their impact on AGA. This approach aimed to elucidate the mechanism of action of QHJR in AGA.

AMP-activated protein kinase (AMPK), the highly conserved serine/threonine-protein kinase, serves as an intracellular energy sensor with significant effects on catabolism and anabolism regulation (Li and Chen, 2019). Referred to as the metabolic master switch, AMPK plays a pivotal role in controlling cellular energy supply and is synthesized in various organs, including the liver, brain, fat cells, and muscle cells (Li and Chen, 2019). Functioning as a regulator of autophagy, AMPK promotes autophagy and facilitates the autophagic degradation of mitochondria (mitophagy) by inducing the fragmentation of damaged mitochondria and promoting the translocation of the autophagy machinery to these damaged organelles (Li and Chen, 2019). AMPK plays a significant role in the maintenance of cellular energy homeostasis, which is closely related to autophagy (Wang S. et al., 2022). AMPK-mediated pathways are involved in autophagy and aging processes (Ge et al., 2022). AMPK can accelerate autophagy through its different actions at diverse autophagy regulatory levels, which are obtained by the specific phosphorylation of autophagy-related protein complexes (Li and Chen, 2019). In summary, AMPK and autophagy are closely related, and AMPK plays a significant role in the regulation of autophagy.

Gout is commonly associated with excesses in soluble urate and in nutrition, both of which involve AMPK activity (Wang et al., 2016; Terkeltaub, 2017). AMPK activation has been found to alleviate high uric acid-induced Na^+^-K^+^-ATPase signaling impairment and cell injury in renal tubules (Xiao et al., 2019). Charles McWherter et al. (McWherter et al., 2018) found that Arhalofenate acid inhibits monosodium urate crystal-induced inflammatory responses through activation of AMPK signaling, which likely contributes to a reduction of gout flares (McWherter et al., 2018). These findings suggest that AMPK may play a role in the development and treatment of gouty arthritis. Further research is needed to fully understand the implications of AMPK in gout and to develop effective treatments targeting AMPK. The activation of AMPK is found to cause autophagy through two distinct mechanisms, namely, the direct phosphorylation of ULK1 (Unc-51-Like Kinase 1, a mammalian orthologue of Atg1) and the inhibition of the mammalian target of rapamycin (mTOR) protein kinase complex (Li and Chen, 2019). This signal pathway has been confirmed, also providing a strong theoretical foundation for QHJR treatment of AGA. In this study, to explore the mechanism of QHJR on AGA, we validated whether the Ampkα1/ulk1/mTOR pathway could be activated by QHJR to regulate autophagy, reduce inflammation, and effectively alleviate AGA.

## 2 Methods

### 2.1 QHJR metabolites screening

We employed the TCMSP database (https://old.tcmsp-e.com/tcmsp.php) (Ru et al., 2014) to explore the metabolite composition of QHJR. This database provides comprehensive information on various molecular aspects, such as composition number, molecular name, molecular weight, fat-water partition coefficient, hydrogen bond donor-acceptor count, oral bioavailability (OB), intestinal epithelium permeability, blood-brain barrier (BBB) permeability, drug similarity (DL), and drug half-life (HL). The OB represents the percentage of unchanged drug that reaches the systemic circulation after oral administration. DL indexes can be used to optimize pharmacokinetic and pharmaceutical properties, such as solubility and chemical stability (Zhou et al., 2020; Liu et al., 2023). The criteria of OB ≥ 30% and DL ≥ 0.18 have been set to screen for active compounds because they indicate that the compound has good oral bioavailability and drug-like properties, which are important for a compound to be effective as a drug (https://www.tcmsp-e.com/load_intro.php?id=29) (Yu Y. et al., 2020; Ruan et al., 2020; Ye et al., 2023).

### 2.2 Network construction

The UniProt database (https://www.uniprot.org/) and Search Tool for the Retrieval of Interacting Genes database (https://string-db.org) were utilized to convert protein gene names and obtain drug component targets. For comprehensive information on human genes, encompassing genome, proteome, transcription, heredity, and function, we turned to the GeneCards Database (https://www.genecards.org/) (Safran et al., 2010). AGA-related targets were gathered from the GeneCards Database. The cross-targets were obtained by merging the disease-related targets with the drug-component targets. After the data were imported into Cytoscape 3.9.1 software (Shannon et al., 2003), a “Botanical drug-metabolite-target” network model was constructed, in which the nodes represent herbs, ingredients, and targets, while the edges represent the relationship role among the three nodes. We calculated the ‘degree’ value according to the number of associations between each node (Liu et al., 2023; Ye et al., 2023).

### 2.3 Data sources and screen out DEGs

The initial step of our study involved performing a keyword search using “acute gouty arthritis” and “*Homo sapiens*” in the search field of the GEO database (https://www.ncbi.nlm.nih.gov/geo/). These keywords were selected to retrieve relevant data. Through this search, we identified gene chip data with the accession number GSE160170. The GSE160170 dataset was based on the GPL21827 platform, specifically the Agilent-079487 Arraystar Human LncRNA microarray V4 (Probe Name version). This dataset comprised a total of 6 specimens from individuals with gout and 6 specimens from individuals serving as normal controls.

### 2.4 PPI network

To construct a protein-protein interaction (PPI) network, we employed the STRING database (https://string-db.org/) (Szklarczyk et al., 2021). Subsequently, the Cytoscape 3.9.1 software’s “cytoHubba” plugin (Doncheva et al., 2023) was utilized to compute the top ten hub genes from both upregulated and downregulated hub genes.

### 2.5 GO and KEGG analysis

To investigate the functional annotations of DEGs, we performed GO analysis (Liu et al., 2018)and KEGG (Kanehisa et al., 2017) analysis using the online tool DAVID (https://david.ncifcrf.gov/). To identify significantly enriched genes, we set the critical value at *p* < 0.05 and |log2(FC)| > 1. In this study, the “clusterprofiler” package in the R software was employed to analyze the primary functions of the DEGs.

### 2.6 Preparation of drugs

11 botanical drugs of QHJR (Supplementary Table S1) were purchased from Shandong Provincial Hospital of TCM pharmacy (Jinan, China), and prepared using the water decoction and alcohol precipitation method at the Experimental Center of Shandong University of TCM (Jinan, China). Colchicine (MedChemExpress, Lot No: HY-16569), 200 mg per unit. Rapamycin (MedChemExpress, Lot No: HY-10219), 10 mg per unit. 3-Methyladenine (MedChemExpress, Lot No: HY-19312), 50 mg per unit.

First, fry the Atractylodis rhizoma and Coicis semen until they turn slightly yellow, and prepare the Plantaginis semen by wrapping it with gauze. Soak all the botanical drugs (except Plantaginis semen) in purified water for 2 h, then remove them and prepare for decoction. Next, place all the botanical drugs (Polygoni cuspidati rhizoma et radix, Spatholobi caulis, Dioscoreae spongiosae rhizoma, Atractylodis rhizoma, Plantaginis semen, Clematidis radix et rhizoma, Smilacis glabrae rhizoma, Poria, Curcumae longae rhizoma, Perillae folium, Coicis semen) in a pot filled with 2 L of water and boil for 0.5 h. Concentrate the decoction to concentrations of 5.15 g/mL (botanical drug/water), 2.56 g/mL (botanical drug/water), and 1.3 g/mL (botanical drug/water) respectively according to the concentration requirements of the QHJR-High, QHJR-Medium, and QHJR-Low groups. The “g/mL” means “drug—solvent ratio”, which is all the botanical drugs of QHJR to pure water. Filter the botanical drugs using gauze to collect the herbal liquid. The decoction was then entrusted to a temperature-controlled refuge within a refrigerator, maintained at a frosty 4 °C.

### 2.7 QHJR extract and HPLC-Q-Orbitrap-MS analysis

Prepare the drug solution (QHJR) (Supplementary Table S1) with a concentration of 2.56 g/mL (botanical drug/water) following the method described above and store it in a refrigerator at −18 °C. After thawing, the samples were vigorously treated by vortexing for 30 s and centrifugation at 12,000 rpm and 4°C for 10 min. A 200 μL portion from each sample was transferred to an Eppendorf tube and combined with 1,000 μL of a 4:1 (v/v) methanol-water extraction solution. This mixture underwent another round of vortexing and centrifugation at 12,000 rpm and 4°C for 10 min. The resulting supernatant was meticulously filtered through a 0.22-μm filter and placed in an autosampler vial for subsequent analysis. The liquid chromatography (LC) separation utilized an HPLC AQ-C18 column (1.8 μm × 150 mm × 2.1 mm, Welch, China) with mobile phases of 0.1% formic acid A) and methanol B). The LC system operated at a flow rate of 300 μL/min, an injection volume of 5 μL, and the following gradient: 0–5 min (2%–20% B), 5–10 min (20%–50% B), 10–15 min (50%–80% B), 15–20 min (80%–95% B), and 20–27 min (95% B) (Supplementary Table S2). An LC system integrated with a Q-Orbitrap mass spectrometer (Thermo Fisher Scientific, United States) played a pivotal role in analyzing the chemical composition of QHJR. The system employed carefully selected parameters including a sheath gas flow rate of 40 Arb, an auxiliary gas flow rate of 15 Arb, a full MS resolution of 70,000, a capillary temperature of 300°C, an MS/MS resolution of 17,500, a spray voltage of 3.2 kV (positive), and a collision energy of 30 in NCE mode. This setup effectively unraveled the intricate molecular constituents. The task of determining the chemical composition of QHJR was entrusted to Wuhan Xavier Biotechnology Co., Ltd., who followed established protocols and methodologies with meticulous care. The detailed information is available in Supplementary Table S2.

### 2.8 Drug-target network

The key metabolites predicted and identified were searched in the PubChem database (https://pubchem.ncbi.nlm.nih.gov/) to obtain their structural information. The obtained structures were then imported into Swiss Target Prediction (http://www.swisstargetprediction.ch/) to predict their target proteins. Disease-related target proteins were retrieved from the GeneCards database (https://www.genecards.org/) (Safran et al., 2010)and filtered for those related to the disease among the identified metabolite targets. The disease-related metabolites and their corresponding targets were imported into Cytoscape (Shannon et al., 2003)3.9.1 for constructing a metabolite-target network and analysis.

### 2.9 Animals and establishment of AGA models

A total of 88 male Sprague-Dawley rats with SPF-grade, weighing 180 ± 20 g, were sourced from Beijing Weitong Lihua Laboratory Animal Technology Co., Ltd (License number: Beijing Baishan SCXK 2016–0006). The rats were categorized into 8 groups (n = 11) for further experimentation, including the Normal group, Model group, QHJR-H group, QHJR-M group, QHJR-L group, Colchicine group, RAPA g, group, and 3-MA group. The rate in the Normal group injected normal saline (NS) was used as the control. The others were injected with normal MSU to the sestablishAGA model (according to Fischer, Brusco et al. (Fischer et al., 2018) method) on day 5. Normal group and Model group with pure water by gavage, colchicine group with colchicine by gavage. QHJR groups were given QHJR-H, QHJR-M, and QHJR-L, respectively. The 3-MA and RAPA groups were intraperitoneally injected with 3-MA and RAPA, respectively.

### 2.10 H&E assay

The knee joints of rats were dissected to obtain synovial tissues, which were subsequently fixed in 4% paraformaldehyde for a duration exceeding 24 h. The fixed tissues were then embedded in paraffin. Additional synovial tissues were preserved in liquid nitrogen at −80 °C for subsequent Quantitative Real-Time PCR and Western blot assays. Following this, 4-μm thick sections of paraffin-embedded tissue were deparaffinized, stained with hematoxylin and eosin (HE), subjected to dehydration using fractional xylene and ethanol, and ultimately sealed with neutral gums.

### 2.11 ELISA assay

Blood samples were collected from the abdominal aorta of rats, and the freshly obtained blood was allowed to stand before being centrifuged at 3500 rpm for a duration of 10 min. The serum was extracted at −80 °C for preservation. The levels of TNF-α, IL-1β, and IL-6 were determined using an ELISA kit, following the manufacturer’s instructions.

### 2.12 qRT-PCR assay

Ex Taq-enzyme Kit (Solarbio PC1100) was employed to extract total synovial RNA, while PrimeScript RTregent Kit (Takara, Japan) was used to prepare cDNA through reverse transcription. qRT-PCR machine CFX96 (Excell Bio IT041-0002, China) was used for qRT-PCR, and the reaction system included respective primers (1 μL each), Power SYBR^®^ Green Master Mix (2*, 12.5 μL), DEPC water (9.5 μL) and cDNA (1 μL). The reaction procedure was presented as follows activation under 95°C; 20-s amplification under 95°C; 30-s un3058°C and 20-s under 72°C for a total of 40 cycles. The 2^−ΔΔCT^ method was performed to quantify PCR analysis. Supplementary Table S3 displays the sequences of all primers utilized in this study (Sangon Biotech, Shanghai, China).

### 2.13 Western blot

The mixture containing RIPA lysates and protease/phosphatase inhibitors was adopted for extracting total synovial proteins. Protein content was quantified by a BCA kit (Solarbio PC0020). Protein aliquots were separated through SDS-PAGE, followed by transfer onto PVDF membranes (Millipore). Then, membranes were blocked using 5% defatted milk for 2 h, followed by overnight primary antibody incubation under 4°C (as shown in Supplementary Table S4). Next, the ECL Kit (Solarbio, no. PE0010) was used for visualizing protein bands, using β-actin as the endogenous control. Images were analyzed with ImageJ (Table 4).

### 2.14 Statistical analysis

There are 3 samples for each set of data. Data were represented by means ± SD. Tukey’s multiple comparison test was conducted by one-way ANOVA by GraphPad Prism version 8.0. *p < 0.05,**p < 0.01, ***p < 0.001 represented obviously difference.

## 3 Results

### 3.1 QHJR’s target prediction for AGA

A comprehensive search for 1,620 metabolites in QHJR was performed using the TCMSP database, including 315 from Polygoni Cuspidati Rhizoma Et Radix, 405 from Spatholobus Suberectus Dunn, 14 from Dioscoreae Septemlo Bae Rhizoma, 67 from Atractylodes lancea (Thunb.)Dc., 189 from Plantaginis Semen, 67 from Radix Clematidis, 315 from Smilacis Glabrae Rhixoma, 21 from Poria Cocos (Schw.) Wolf., 38 from Curcumaelongae Rhizoma, 145 from Perilla Frutescens, and 44 from Coicis Semen. Screening based on OB ≥ 30% and DL ≥ 0.18 yielded 117 metabolites, with 10 from Polygoni Cuspidati Rhizoma Et Radix, 24 from Spatholobus Suberectus Dunn, 2 from Dioscoreae Septemlo Bae Rhizoma, 9 from Atractylodes Lancea (Thunb.)Dc., 9 from Plantaginis Semen, 7 from Radix Clematidis, 15 from Smilacis Glabrae Rhixoma, 15 from Poria Cocos (Schw.) Wolf., 3 from Curcumaelongae Rhizoma, 14 from Perilla Frutescens, and 9 from Coicis Semen. After removing duplicates, 94 metabolites were identified. Table 1 displays these metabolites found in QHJR.

**TABLE 1:** Metabolites were predicted of QHJR in the TCMSP database.

Botanical drug	Code	Metabolites	OB(%)	DL
Polygoni Cuspidati Rhizoma Et Radix	MOL013281	6,8-Dihydroxy-7-methoxyxanthone	35.83	0.21
Polygoni Cuspidati Rhizoma Et Radix	MOL013287	Physovenine	106.21	0.19
Polygoni Cuspidati Rhizoma Et Radix	MOL013288	Picralinal	58.01	0.75
Polygoni Cuspidati Rhizoma Et Radix	MOL002259	Physciondiglucoside	41.65	0.63
Polygoni Cuspidati Rhizoma Et Radix	MOL002268	rhein	47.07	0.28
Polygoni Cuspidati Rhizoma Et Radix	MOL002280	Torachrysone-8-O-beta-D-(6'-oxayl)-glucoside	43.02	0.74
Polygoni Cuspidati Rhizoma Et Radix	MOL000358	beta-sitosterol	36.91	0.75
Polygoni Cuspidati Rhizoma Et Radix	MOL000492	(+)-catechin	54.83	0.24
Polygoni Cuspidati Rhizoma Et Radix	MOL000006	luteolin	36.16	0.25
Polygoni Cuspidati Rhizoma Et Radix	MOL000098	quercetin	46.43	0.28
Spatholobus Suberectus Dunn	MOL000296	hederagenin	36.91	0.75
Spatholobus Suberectus Dunn	MOL000033	(3S,8S,9S,10R,13R,14S,17R)-10,13-dimethyl-17-[(2R,5S)-5-propan-2-yloctan-2-yl]-2,3,4,7,8,9,11,12,14,15,16,17-dodecahydro-1H-cyclopenta [a]phenanthren-3-ol	36.23	0.78
Spatholobus Suberectus Dunn	MOL000392	formononetin	69.67	0.21
Spatholobus Suberectus Dunn	MOL000417	Calycosin	47.75	0.24
Spatholobus Suberectus Dunn	MOL000449	Stigmasterol	43.83	0.76
Spatholobus Suberectus Dunn	MOL000461	3,7-dihydroxy-6-methoxy-dihydroflavonol	43.8	0.26
Spatholobus Suberectus Dunn	MOL000468	8-o-Methylreyusi	70.32	0.27
Spatholobus Suberectus Dunn	MOL000469	3-Hydroxystigmast-5-en-7-one	40.93	0.78
Spatholobus Suberectus Dunn	MOL000470	8-C-α-L-arabinosylluteolin	35.54	0.66
Spatholobus Suberectus Dunn	MOL000471	aloe-emodin	83.38	0.24
Spatholobus Suberectus Dunn	MOL000483	(Z)-3-(4-hydroxy-3-methoxy-phenyl)-N-[2-(4-hydroxyphenyl)ethyl]acrylamide	118.35	0.26
Spatholobus Suberectus Dunn	MOL000490	petunidin	30.05	0.31
Spatholobus Suberectus Dunn	MOL000491	Augelicin	37.5	0.66
Spatholobus Suberectus Dunn	MOL000493	campesterol	37.58	0.71
Spatholobus Suberectus Dunn	MOL000497	licochalcone a	40.79	0.29
Spatholobus Suberectus Dunn	MOL000500	Vestitol	74.66	0.21
Spatholobus Suberectus Dunn	MOL000501	Consume close grain	68.12	0.27
Spatholobus Suberectus Dunn	MOL000502	Cajinin	68.8	0.27
Spatholobus Suberectus Dunn	MOL000503	Medicagol	57.49	0.6
Spatholobus Suberectus Dunn	MOL000506	Lupinidine	61.89	0.21
Spatholobus Suberectus Dunn	MOL000507	Psi-Baptigenin	70.12	0.31
Dioscoreae Septemlo Bae Rhizoma	MOL013233	EINECS 213–897–0	71.96	0.72
Dioscoreae Septemlo Bae Rhizoma	MOL000546	diosgenin	80.88	0.81
Atractylodes Lancea (Thunb.)Dc.	MOL000173	wogonin	30.68	0.23
Atractylodes Lancea (Thunb.)Dc.	MOL000179	2-Hydroxyisoxypropyl-3-hydroxy-7-isopentene-2,3-dihydrobenzofuran-5-carboxylic	45.2	0.2
Atractylodes Lancea (Thunb.)Dc.	MOL000184	NSC63551	39.25	0.76
Atractylodes Lancea (Thunb.)Dc.	MOL000186	Stigmasterol 3-O-beta-D-glucopyranoside_qt	43.83	0.76
Atractylodes Lancea (Thunb.)Dc.	MOL000188	3β-acetoxyatractylone	40.57	0.22
Atractylodes Lancea (Thunb.)Dc.	MOL000085	beta-daucosterol_qt	36.91	0.75
Atractylodes Lancea (Thunb.)Dc.	MOL000088	beta-sitosterol 3-O-glucoside_qt	36.91	0.75
Atractylodes Lancea (Thunb.)Dc.	MOL000092	daucosterin_qt	36.91	0.76
Atractylodes Lancea (Thunb.)Dc.	MOL000094	daucosterol qt	36.91	0.76
Plantaginis Semen	MOL001663	(4aS,6aR,6aS,6bR,8aR,10R,12aR,14bS)-10-hydroxy-2,2,6a,6b,9,9,12a-heptamethyl-1,3,4,5,6,6a,7,8,8a,10,11,12,13,14b-tetradecahydropicene-4a-carboxylic acid	32.03	0.76
Plantaginis Semen	MOL001735	Dinatin	30.97	0.27
Plantaginis Semen	MOL000359	sitosterol	36.91	0.75
Plantaginis Semen	MOL005869	daucostero_qt	36.91	0.75
Plantaginis Semen	MOL007813	Dihydrotricetin	58.12	0.28
Plantaginis Semen	MOL007819	Hypolaetin	33.24	0.28
Plantaginis Semen	MOL007835	orobanchoside_qt	55.99	0.82
Plantaginis Semen	MOL007836	plantaginin_qt	54.04	0.24
Radix Clematidis	MOL002372	(6Z,10E,14E,18E)-2,6,10,15,19,23-hexamethyltetracosa-2,6,10,14,18,22-hexaene	33.55	0.42
Radix Clematidis	MOL005594	ClematosideA'_qt	37.51	0.76
Radix Clematidis	MOL005598	Embinin	33.91	0.73
Radix Clematidis	MOL005603	Heptyl phthalate	42.26	0.31
Smilacis Glabrae Rhixoma	MOL013117	4,7-Dihydroxy-5-methoxyl-6-methyl-8-formyl-flavan	37.03	0.28
Smilacis Glabrae Rhixoma	MOL013118	Neoastilbin	40.54	0.74
Smilacis Glabrae Rhixoma	MOL013119	Enhydrin	40.56	0.74
Smilacis Glabrae Rhixoma	MOL013129	(2R,3R)-2-(3,5-dihydroxyphenyl)-3,5,7-trihydroxychroman-4-one	63.17	0.27
Smilacis Glabrae Rhixoma	MOL001736	(-)-taxifolin	60.51	0.27
Smilacis Glabrae Rhixoma	MOL004328	naringenin	59.29	0.21
Smilacis Glabrae Rhixoma	MOL004567	isoengelitin	34.65	0.7
Smilacis Glabrae Rhixoma	MOL004575	astilbin	36.46	0.74
Smilacis Glabrae Rhixoma	MOL004576	taxifolin	57.84	0.27
Smilacis Glabrae Rhixoma	MOL004580	cis-Dihydroquercetin	66.44	0.27
Smilacis Glabrae Rhixoma	MOL000273	(2R)-2-[(3S,5R,10S,13R,14R,16R,17R)-3,16-dihydroxy-4,4,10,13,14-pentamethyl-2,3,5,6,12,15,16,17-octahydro-1H-cyclopenta [a]phenanthren-17-yl]-6-methylhept-5-enoic acid	46.43	0.28
Poria Cocos (Schw.) Wolf.	MOL000275	trametenolic acid	30.93	0.81
Poria Cocos (Schw.) Wolf.	MOL000276	7,9 (11)-dehydropachymic acid	38.71	0.8
Poria Cocos (Schw.) Wolf.	MOL000279	Cerevisterol	35.11	0.81
Poria Cocos (Schw.) Wolf.	MOL000280	(2R)-2-[(3S,5R,10S,13R,14R,16R,17R)-3,16-dihydroxy-4,4,10,13,14-pentamethyl-2,3,5,6,12,15,16,17-octahydro-1H-cyclopenta [a]phenanthren-17-yl]-5-isopropyl-hex-5-enoic acid	37.96	0.77
Poria Cocos (Schw.) Wolf.	MOL000282	ergosta-7,22E-dien-3beta-ol	31.07	0.82
Poria Cocos (Schw.) Wolf.	MOL000283	Ergosterol peroxide	43.51	0.72
Poria Cocos (Schw.) Wolf.	MOL000285	(2R)-2-[(5R,10S,13R,14R,16R,17R)-16-hydroxy-3-keto-4,4,10,13,14-pentamethyl-1,2,5,6,12,15,16,17-octahydrocyclopenta [a]phenanthren-17-yl]-5-isopropyl-hex-5-enoic acid	40.36	0.81
Poria Cocos (Schw.) Wolf.	MOL000287	3beta-Hydroxy-24-methylene-8-lanostene-21-oic acid	38.26	0.82
Poria Cocos (Schw.) Wolf.	MOL000289	pachymic acid	38.7	0.81
Poria Cocos (Schw.) Wolf.	MOL000290	Poricoic acid A	33.63	0.81
Poria Cocos (Schw.) Wolf.	MOL000291	Poricoic acid B	30.61	0.76
Poria Cocos (Schw.) Wolf.	MOL000292	poricoic acid C	30.52	0.75
Poria Cocos (Schw.) Wolf.	MOL000300	dehydroeburicoic acid	38.15	0.75
Poria Cocos (Schw.) Wolf.	MOL000953	CLR	44.17	0.83
Curcumaelongae Rhizoma	MOL005030	gondoic acid	37.87	0.68
Perilla Frutescens	MOL006202	LAX	30.7	0.2
Perilla Frutescens	MOL002773	beta-carotene	44.11	0.2
Perilla Frutescens	MOL006209	cyanin	37.18	0.58
Perilla Frutescens	MOL006210	eugenyl-β-D-glucopyranoside (cirtrusinc)	47.42	0.76
Perilla Frutescens	MOL001506	Supraene	40.52	0.23
Perilla Frutescens	MOL001749	ZINC03860434	33.55	0.42
Perilla Frutescens	MOL001771	poriferast-5-en-3beta-ol	43.59	0.35
Perilla Frutescens	MOL007179	Linolenic acid ethyl ester	36.91	0.75
Perilla Frutescens	MOL007514	methyl icosa-11,14-dienoate	46.1	0.2
Perilla Frutescens	MOL001323	Sitosterol alpha1	39.67	0.23
Coicis Semen	MOL001494	Mandenol	43.28	0.78
Coicis Semen	MOL002882	[(2R)-2,3-dihydroxypropyl] (Z)-octadec-9-enoate	42	0.19
Coicis Semen	MOL008118	Coixenolide	34.13	0.3
Coicis Semen	MOL008121	2-Monoolein	32.4	0.43

**FIGURE 1:**
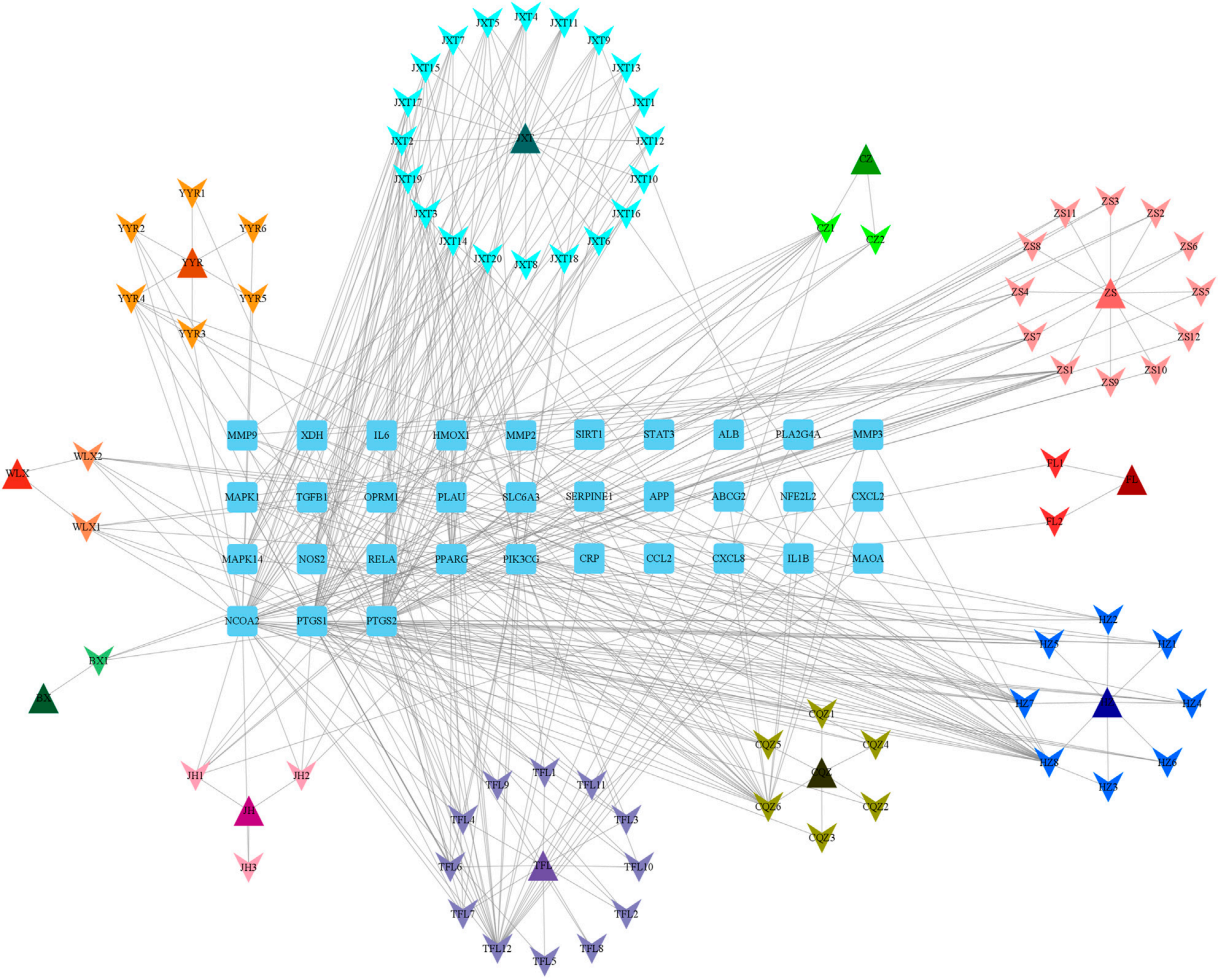
Network of QHJR’s active-metabolite-target. The square represents the target. The triangle represents the traditional Chinese medicine in QHJR. The arrow represents the active metabolite.

Protein gene names were converted using the STRING and UniProt databases, and AGA targets were subsequently identified in GeneCards. The intersection of these targets was then employed to construct the “Botanical drug-Metabolite-Target” network, as depicted in Figure 1. Our predictions suggested that these metabolites predicted in QHJR may have the potential to impact the target, as detailed in Table 2.

**TABLE 2:** The Metabolites of botanical drugs in QHJR for AGA by predicting.

Metabolites	ID	Degree	AverageShortestPathLength	BetweennessCentrality	ClosenessCentrality
quercetin	MOL000098	25	1.965811966	0.07219322	0.508695652
luteolin	MOL000006	14	2.136752137	0.026107878	0.468
wogonin	MOL000173	11	2.427350427	0.023736504	0.411971831
licochalcone a	MOL000497	11	2.205128205	0.030326409	0.453488372
formononetin	MOL000392	9	2.478632479	0.021283568	0.403448276
naringenin	MOL004328	7	2.478632479	0.003962642	0.403448276
Hypolaetin	MOL007819	7	2.273504274	0.008043303	0.439849624
Psi-Baptigenin	MOL000507	7	2.495726496	0.003644405	0.400684932
Cajinin	MOL000502	7	2.273504274	0.006374379	0.439849624
Vestitol	MOL000500	7	2.495726496	0.004189551	0.400684932
8-o-Methylreyusi	MOL000468	7	2.273504274	0.006374379	0.439849624
Stigmasterol	MOL000449	7	2.273504274	0.009350993	0.439849624
Calycosin	MOL000417	7	2.273504274	0.006374379	0.439849624
beta-sitosterol	MOL000358	7	2.256410256	0.009819465	0.443181818
Dinatin	MOL001735	6	2.290598291	0.006881825	0.436567164
petunidin	MOL000490	6	2.290598291	0.005380608	0.436567164
aloe-emodin	MOL000471	6	2.290598291	0.006377143	0.436567164
Physovenine	MOL013287	6	2.512820513	0.006369406	0.397959184
beta-carotene	MOL002773	5	2.495726496	0.019538023	0.400684932
taxifolin	MOL004576	5	2.512820513	0.002066119	0.397959184
campesterol	MOL000493	5	2.307692308	0.003231239	0.433333333
rhein	MOL002268	5	2.290598291	0.004853717	0.436567164
6,8-Dihydroxy-7-methoxyxanthone	MOL013281	5	2.512820513	0.003512472	0.397959184
Mandenol	MOL001494	4	2.341880342	0.006668133	0.427007299
cis-Dihydroquercetin	MOL004580	4	2.52991453	0.001265361	0.39527027
(-)-taxifolin	MOL001736	4	2.52991453	0.001265361	0.39527027
4,7-Dihydroxy-5-methoxyl-6-methyl-8-formyl-flavan	MOL013117	4	2.52991453	0.001265361	0.39527027
Dihydrotricetin	MOL007813	4	2.512820513	0.002609127	0.397959184
3β-acetoxyatractylone	MOL000188	4	2.564102564	0.006458836	0.39
diosgenin	MOL000546	4	2.581196581	0.025255951	0.387417219
hederagenin	MOL000296	4	2.324786325	0.002425875	0.430147059
(+)-catechin	MOL000492	4	2.324786325	0.003898026	0.430147059
Linolenic acid ethyl ester	MOL007179	3	2.52991453	0.001435086	0.39527027
LAX	MOL006202	3	2.52991453	0.001435086	0.39527027
gondoic acid	MOL005030	3	2.495726496	0.001747611	0.400684932
(2R,3R)-2-(3,5-dihydroxyphenyl)-3,5,7-trihydroxychroman-4-one	MOL013129	3	2.564102564	0.000825	0.39
Consume close grain	MOL000501	3	2.564102564	0.000467	0.39
(Z)-3-(4-hydroxy-3-methoxy-phenyl)-N-[2-(4-hydroxyphenyl)ethyl]acrylamide	MOL000483	3	2.564102564	0.000467	0.39
3,7-dihydroxy-6-methoxy-dihydroflavonol	MOL000461	3	2.564102564	0.000467	0.39
2-Monoolein	MOL008121	2	2.905982906	0.00122972	0.344117647
Sitosterol alpha1	MOL001323	2	2.564102564	0.002295637	0.39
methyl icosa-11,14-dienoate	MOL007514	2	2.820512821	0.000706	0.354545455
poriferast-5-en-3beta-ol	MOL001771	2	2.820512821	0.000706	0.354545455
eugenyl-β-D-glucopyranoside (cirtrusinc)	MOL006210	2	2.547008547	0.000805	0.39261745
cyanin	MOL006209	2	2.547008547	0.000805	0.39261745
CLR	MOL000953	2	2.923076923	0.001725953	0.342105263
(2R)-2-[(3S,5R,10S,13R,14R,16R,17R)-3,16-dihydroxy-4,4,10,13,14-pentamethyl-2,3,5,6,12,15,16,17-octahydro-1H-cyclopenta [a]phenanthren-17-yl]-6-methylhept-5-enoic acid	MOL000273	2	2.923076923	0.008473327	0.342105263
isoengelitin	MOL004567	2	2.598290598	0.000448	0.384868421
daucostero_qt	MOL005869	2	2.905982906	0.000753	0.344117647
sitosterol	MOL000359	2	2.905982906	0.000753	0.344117647
Medicagol	MOL000503	2	2.991452991	0.00014	0.334285714
8-C-α-L-arabinosylluteolin	MOL000470	2	2.598290598	0.000263	0.384868421
Picralinal	MOL013288	2	3.555555556	0.000151	0.28125

### 3.2 Chemical composition of QHJR extract

**FIGURE 2:**
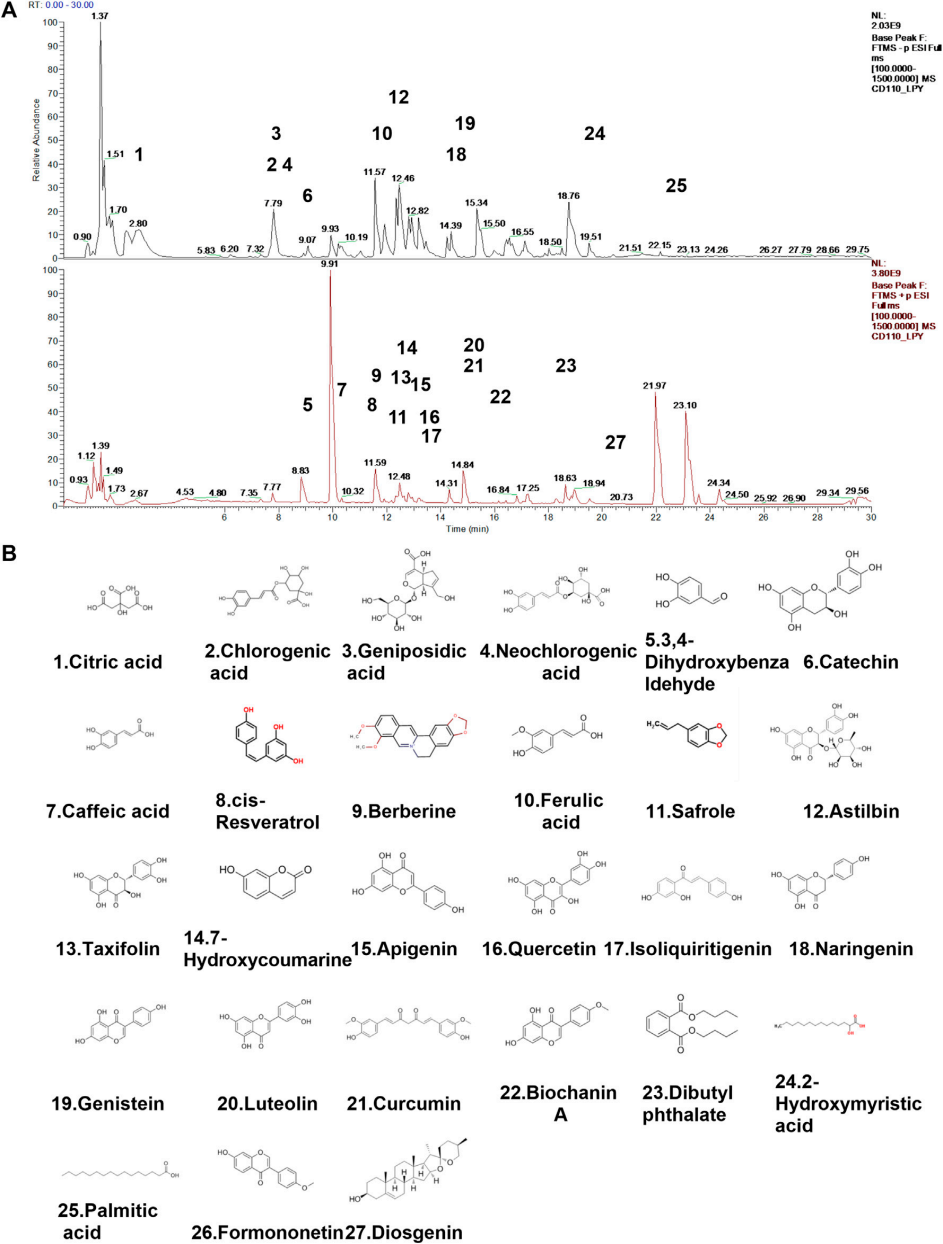
Identification of chemical constituents of QHJR. **(A)** Total ion chromatographic analysis. **(B)** Main metabolite structural formula. The structural formulas of the main metabolites shown in **(B)** are sourced from the websites (https://hmdb.ca/metabolites/HMDB0000002) and (https://www.chemsrc.com/).

The main components in extracts of QHJR were analyzed qualitatively based on HPLC-Q-Orbitrap-MS. The analysis results are shown in Figure 2 and Table 3, including the total ion chromatogram of HPLC-Q-Orbitrap-MS, as well as the structural formula of the main chemical components in QHJR. According to the above analysis, as well as the comparison of TCMSP database and literature, 27 main metabolites were detected, identified and speculated within 30 min by the mass spectrum behavior and fragment ion characteristics (Figure 2) (Table 3), as follow: Citric acid, Chlorogenic acid, Geniposidic acid, Neochlorogenic acid, 3,4-Dihydroxybenzaldehyde, Catechin, Caffeic acid, cis-Resveratrol, Berberine, Ferulic acid, Safrole, Astilbin, Taxifolin, 7-Hydroxycoumarine, Apigenin, Quercetin, Isoliquiritigenin, Naringenin, Genistein, Luteolin, Curcumin, Biochanin A, Dibutyl phthalate, 2-Hydroxymyristic acid, Palmitic acid, Formononetin, Diosgenin. Of these metabolites, six were found to agree with prior predictions by Network Analysis (quercetin, luteolin, formononetin, naringenin, taxifolin, and diosgenin), while five metabolites (Neochlorogenic acid, Caffeic acid, Berberine, Isoliquiritigenin, Formononetin) of them that are shown in the TCMSP database to not belong to any single herbal component of QHJR. From this, we infer that these metabolites may be the result of interactions between different botanical drugs within QHJR, generating new metabolites. These findings suggest that QHJR may not only work through the individual actions of its constituent herbs but also potentially through the synergistic production of new metabolites, contributing to its therapeutic effect on AGA.

**TABLE 3:** QHJR was analyzed qualitatively by HPLC-Q-Orbitrap-MS.

Name	Metabolites	Formula	RT [min]	+	-		Calc.MW	m/z
QHJR-1	Citric acid	C6 H8 O7	2.788		[M-H]-1		192.0262	191.0188
QHJR-2	Chlorogenic acid	C16 H18 O9	7.726		[M-H + HAc]-1		294.0736	353.0875
QHJR-3	Geniposidic acid	C16 H22 O10	7.801		[M-H]-1		374.1209	373.1135
QHJR-4	Neochlorogenic acid	C16 H18 O9	8.254		[M-H-H2O]-1		372.1055	353.0877
QHJR-5	3,4-Dihydroxybenzaldehyde	C7 H6 O3	9.059	[M + H]+1			138.0316	139.0388
QHJR-6	Catechin	C15 H14 O6	9.068		[M-H]-1		290.0788	289.0718
QHJR-7	Caffeic acid	C9 H8 O4	10.323	[M + H]+1			180.0421	181.0494
QHJR-8	cis-Resveratrol	C14 H12 O3	11.585	[M + H]+1			228.0781	229.0854
QHJR-9	Berberine	C20 H18 N O4	11.672	[M + H]+1			335.1153	336.1226
QHJR-10	Ferulic acid	C10 H10 O4	11.85		[M-H]-1		194.0572	193.0498
QHJR-11	Safrole	C10 H10 O2	12.444	[M + H]+1			162.0679	163.0751
QHJR-12	Astilbin	C21 H22 O11	12.475		[M-H]-1		450.1156	449.1083
QHJR-13	Taxifolin	C15 H12 O7	12.487	[M + H]+1			304.0576	305.0648
QHJR-14	7-Hydroxycoumarine	C9 H6 O3	12.611	[M + H]+1			162.0315	163.0387
QHJR-15	Apigenin	C15 H10 O5	13.226	[M + H]+1			270.0524	271.0597
QHJR-16	Quercetin	C15 H10 O7	13.624	[M + H]+1			302.0421	303.0494
QHJR-17	Isoliquiritigenin	C15 H12 O4	13.689	[M + H]+1			256.0731	257.0804
QHJR-18	Naringenin	C15 H12 O5	14.562		[M-H]-1		272.0684	271.0613
QHJR-19	Genistein	C15 H10 O5	15.02		[M-H]-1		270.0529	269.0457
QHJR-20	Luteolin	C15 H10 O6	15.158	[M + H]+1			286.0476	287.0546
QHJR-21	Curcumin	C21 H20 O6	15.166	[M + H]+1			368.1253	369.1325
QHJR-22	Biochanin A	C16 H12 O5	16.147	[M + H]+1			284.0681	285.0752
QHJR-23	Dibutyl phthalate	C16 H22 O4	18.638	[M + H]+1			278.1512	279.1585
QHJR-24	2-Hydroxymyristic acid	C14 H28 O3	19.733		[M-H]-1		244.2036	243.1963
QHJR-25	Palmitic acid	C16 H32 O2	22.71		[M-H]-1		256.24	255.2327
QHJR-26	Formononetin	C16 H12 O4	15.905	[M + H]+1			268.0731	269.0804
QHJR-27	Diosgenin	C27 H42 O3	20.419	[M + H]+1			396.3021	397.3093

### 3.3 Identification of related genes in AGA

**FIGURE 3:**
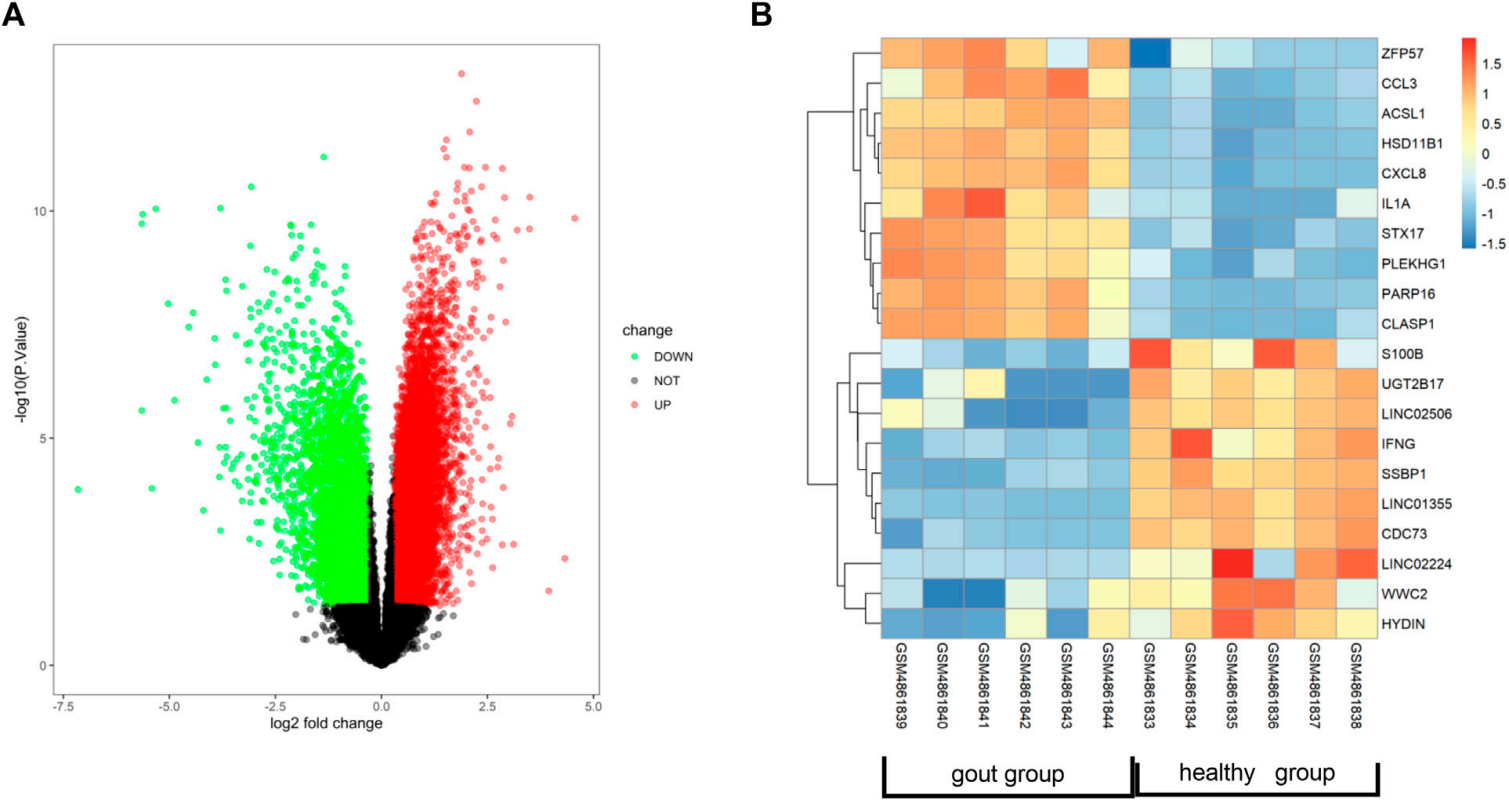
Identification of related genes of AGA. **(A)** Volcano map showing differentially expressed genes in dataset GSE160170. **(B)** Heat map showing gout-related genes in data set GSE160170.

To identify novel targets for treating AGA, we conducted a genetic analysis of this condition. Using the GEO database, we obtained the GSE160170 dataset and performed online analysis using GEO2R. DEGs were screened out as *p* < 0.05 and |log2(FC)| > 1, and it was shown as the volcano (Figure 3A) and heatmap (Figure 3B) by RStudio (Version 4.2.1). By comparing the gout group to the control group, we identified 2,369 DEGs in GSE160170, comprising 1,390 upregulated and 979 downregulated genes. Normalization of the original data was achieved using the R packages ggplot2 (version 3.4.1) and pheatmap (version 1.0.12), enabling visualization of the volcano and hotspot maps depicting the relevant gene expression (Figures 3A,B).

**FIGURE 4:**
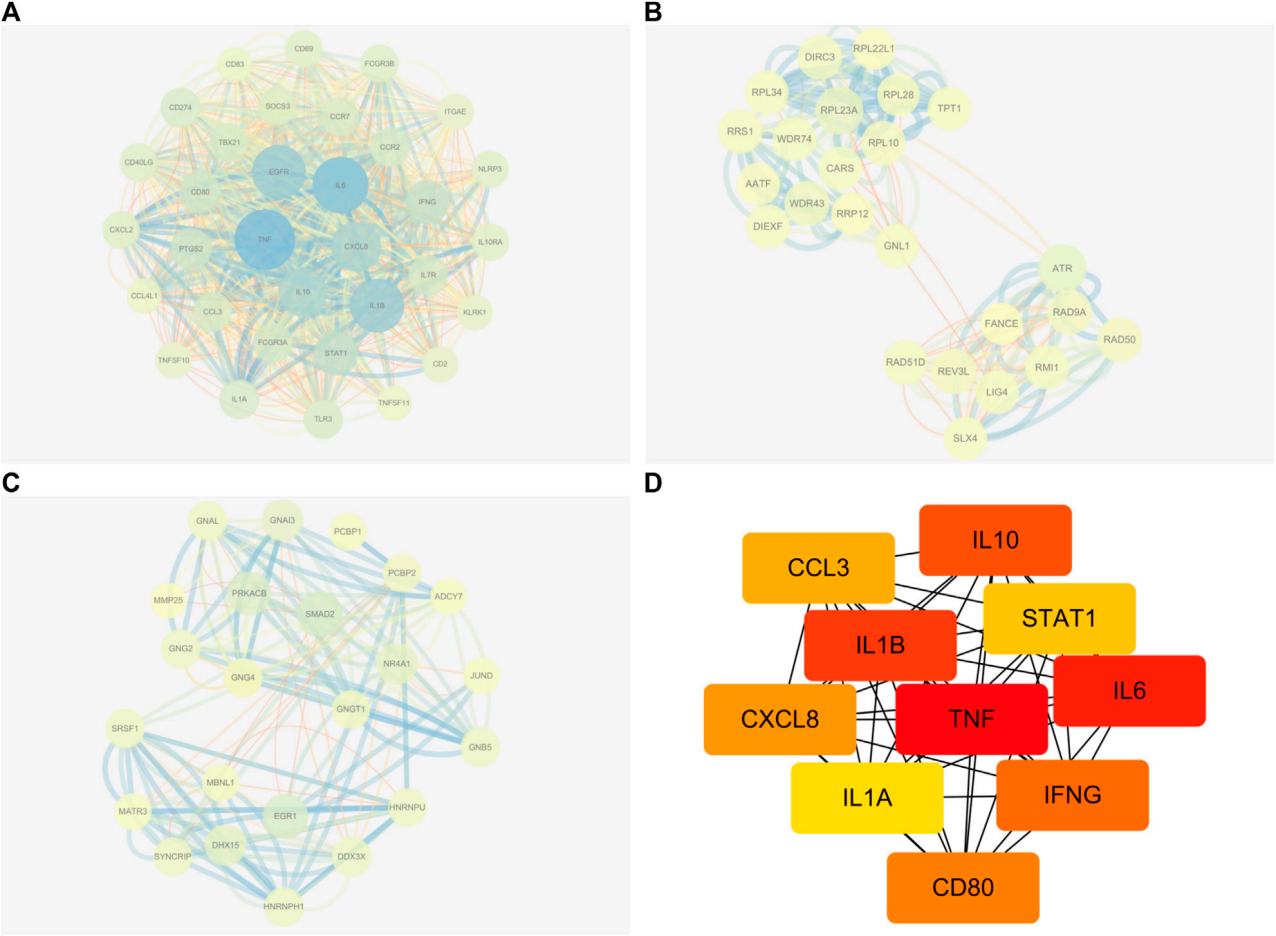
Results of PPI networks analysis of DEGs. **(A-C)** DEGs of PPI network analysis. **(D)** Hub genes of DEGs.

Following the removal of redundant genes, we subjected the remaining 1,412 DEGs from GSE160170 to PPI analysis using the STRING database (Fig. A–C). Then, the top 10 hub genes were identified based on their node degree and included TNF, IL6, IL1B, IL10, IFNG, CD80, CXCL8, CCL3, STAT1, and IL1A (Figure 4D).

### 3.4 KEGG and GO enrichment analysis

**FIGURE 5:**
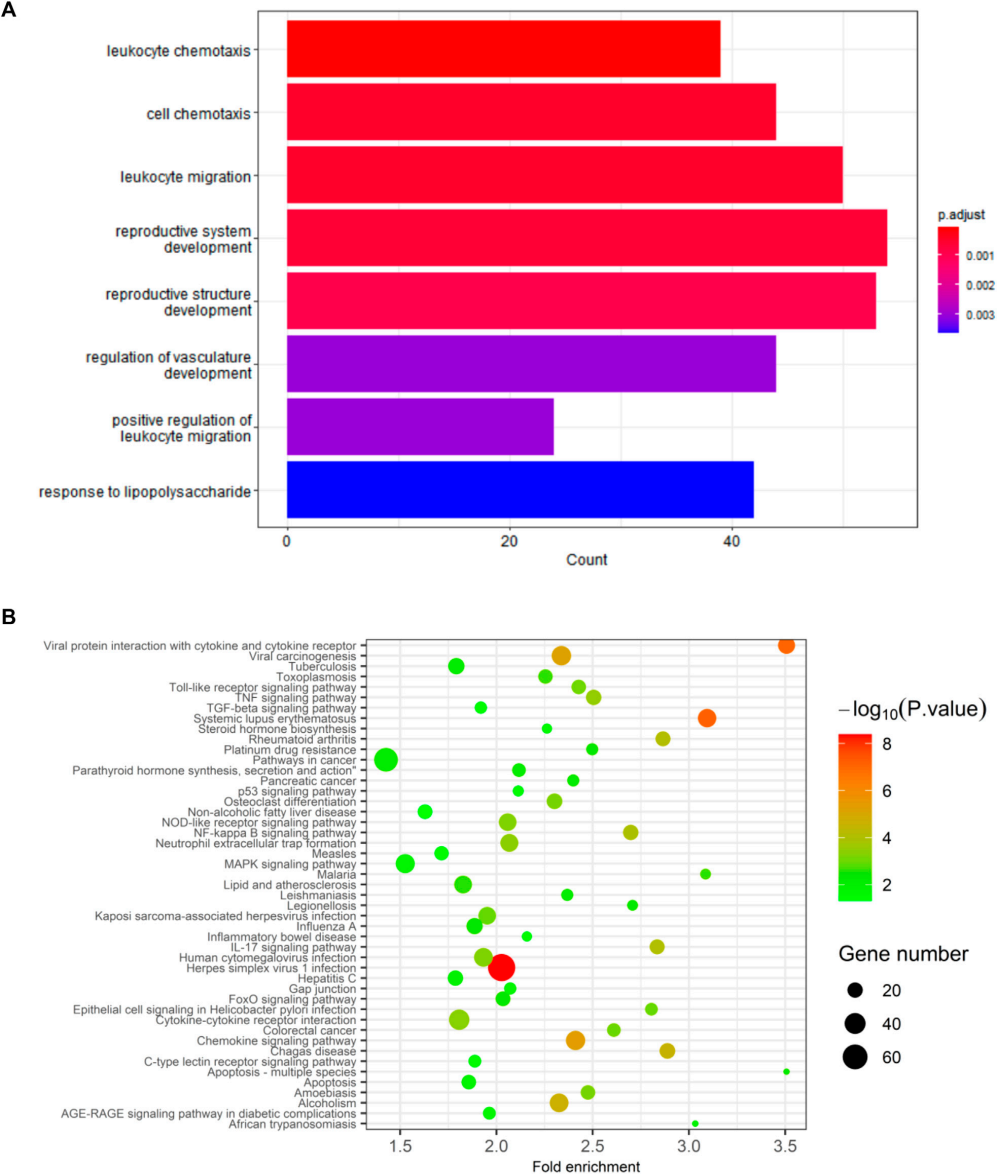
Results of GO and KEGG analysis. **(A)** GO analysis. **(B)** KEGG analysis.

DEGs in GSE160170 exhibit associations with leukocyte chemotaxis, cell chemotaxis, leukocyte migration, reproductive system development, reproductive structure development, regulation of vasculature development, positive regulation of leukocyte migration, *etc.*, based on GO analysis (as depicted in Figure 5A). Furthermore, the DEGs are relevant to various pathways including Viral protein interaction with cytokine and cytokine receptor, Toll-like receptor signaling pathway, TNF signaling pathway, TGF-beta signaling pathway, Rheumatoid arthritis, p53 signaling pathway, Osteoclast differentiation, Non-alcoholic fatty liver disease, NOD-like receptor signaling pathway, NF-kappa B signaling pathway, Neutrophil extracellular trap formation, MAPK signaling pathway, Lipid and atherosclerosis, Inflammatory bowel disease, IL-17 signaling pathway, Hepatitis C, Gap junction, FoxO signaling pathway, Cytokine-cytokine receptor interaction, Chemokine signaling pathway, C-type lectin receptor signaling pathway, Apoptosis-multiple species, Apoptosis, *etc.*, based on KEGG analysis (Figure 5B).

Our current study results, in combination with previous protein-protein interaction (PPI) analysis, indicate that QHJR may reduce inflammation through the involvement of TNF, IL6, IL1B, and other factors. Therefore, we further investigated and evaluated the role of QHJR in the treatment of AGA by establishing a rat model of AGA *in vivo* to explore if it was related to inflammation.

### 3.5 QHJR affects AGA

**FIGURE 6:**
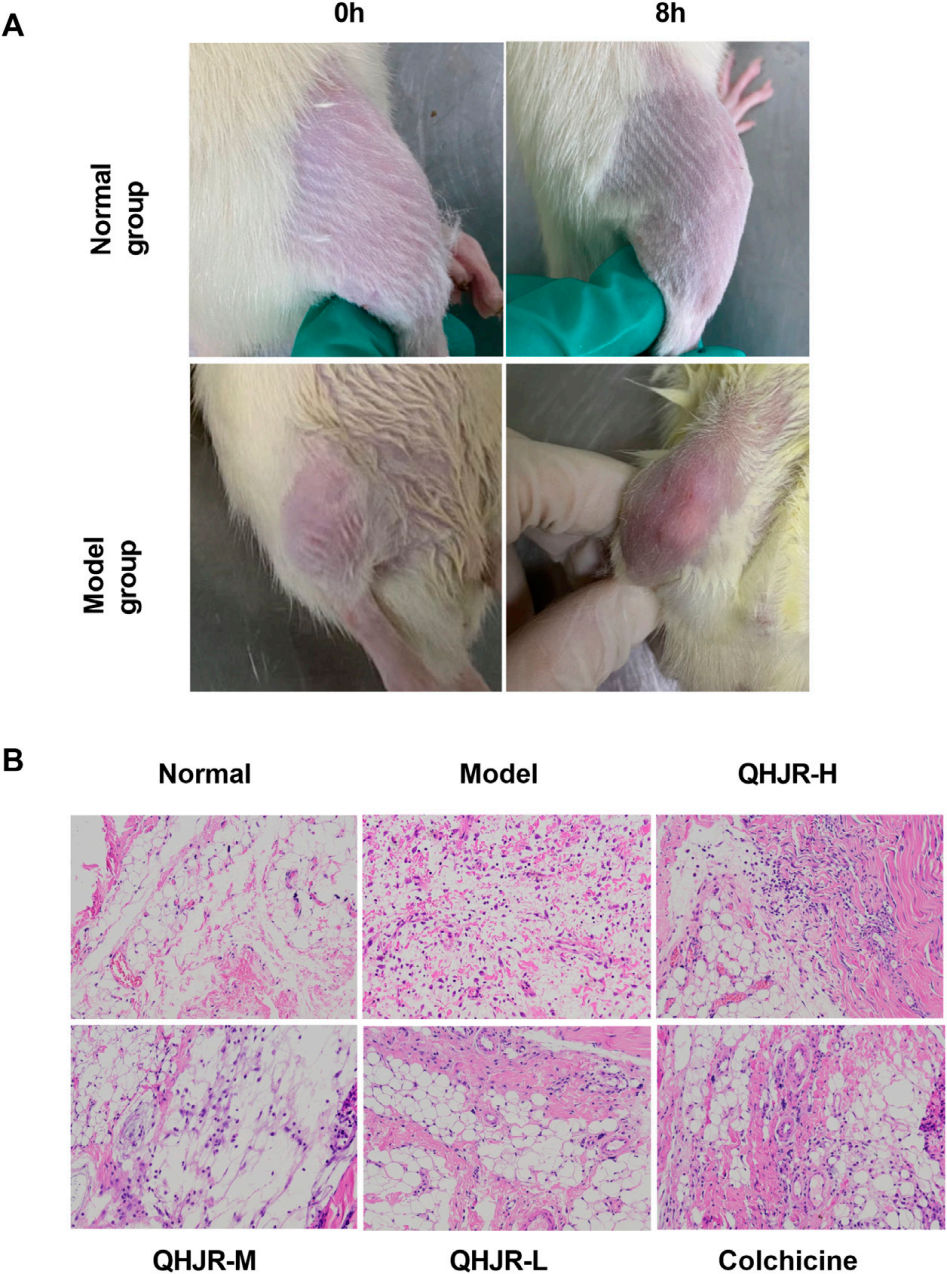
AGA was induced by MSU. **(A)** The swelling of rat knees at 0 and 8 h by MSU injection in Normal and Model groups. **(B)** Synovial tissues were examined by H&E staining.

Firstly, We established the AGA model. Rats in the Normal group were injected with NS, and in other groups were injected with MSU. The previous studies (Fischer et al., 2018; Zhao J. et al., 2022; Wang X. et al., 2022; Zhao L. et al., 2022) have demonstrated that 8 h after the MSU injection is the key point for AGA Model. We observed the swelling of rat knees, with few changes in the Normal control group, while it had obvious redness and swelling in the Model group (Figure 6A), suggesting that the AGA model was established successfully by MSU injection. H&E assay revealed that the lining cells of the synovial membrane were regularly arranged in a single layer, and the surface of the synovial membrane was smooth and orderly without inflammatory infiltration in Normal group (Figure 6B). As displayed in the Model group, it was characterized by several inflammatory cells infiltration (including lymphocytes, plasma cells, neutrophils, and eosinophils.) (Figure 6B). The observed results are in line with the successful outcome of the model construction. Notably, both the QHJR and colchicine groups exhibited varying degrees of alleviation in symptoms associated with AGA, such as inflammatory cell infiltration. These findings suggest that QHJR could effectively mitigate AGA in a manner comparable to that of colchicine.

### 3.6 The main potential targets of QHJR

**FIGURE 7:**
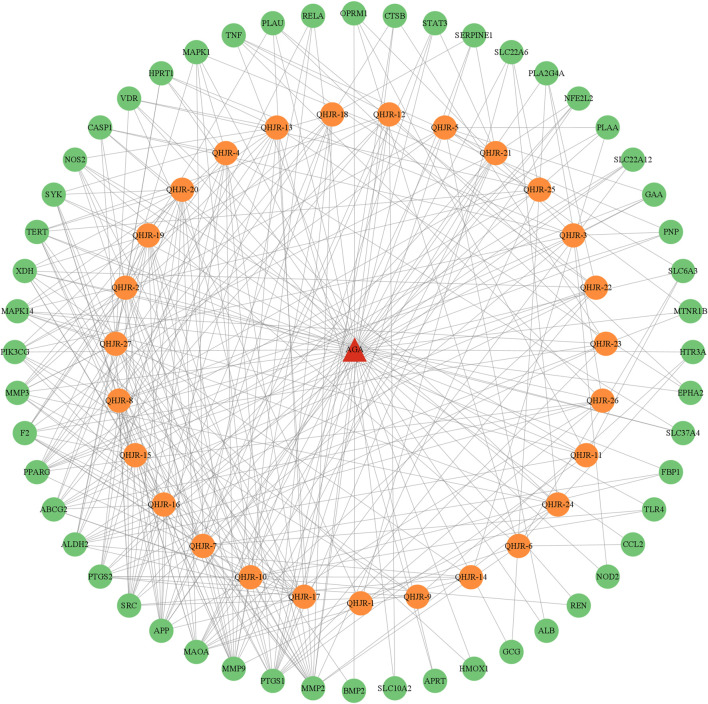
Network of drug targets.

As depicted in Figure 7, the multi-component traditional Chinese medicine QHJR exhibits therapeutic effects on AGA through a multi-target mechanism, as evidenced by the ability of 27 chemical components to act on diverse targets. Through comprehensive network analysis, the top 6 metabolites, namely, QHJR-10 (Caffeic acid), QHJR-17 (cis-Resveratrol), QHJR-7 (Ferulic acid), QHJR-8 (Apigenin), QHJR-15 (Quercetin), and QHJR-16 (Isoliquiritigenin), were identified with degrees of 16, 16, 15, 13, 13, and 13, respectively, implying they may be the key potential metabolites of QHJR for anti-AGA (Table 4). It was shown that 2 metabolites of them, which is QHJR-10 (Caffeic acid) and QHJR-16 (Isoliquiritigenin), as shown in the TCMSP database, do not belong to any single herbal component of QHJR. This suggests that QHJR may not be replaceable by any single botanical drug. However, further validation is still required to prove the research’s accuracy.

**TABLE 4:** Network topology parameters of metabolites in QHJR.

Name	Degree	AverageShortestPathLength	BetweennessCentrality	ClosenessCentrality
QHJR-10	16	2.303797468	0.025338657	0.434065934
QHJR-17	16	2.278481013	0.022804202	0.438888889
QHJR-7	15	2.329113924	0.020040591	0.429347826
QHJR-8	13	2.35443038	0.017043398	0.424731183
QHJR-15	13	2.35443038	0.010956889	0.424731183
QHJR-16	13	2.35443038	0.011578714	0.424731183

### 3.7 Anti-inflammation is the vital way to anti-AGA for QHJR

**FIGURE 8:**
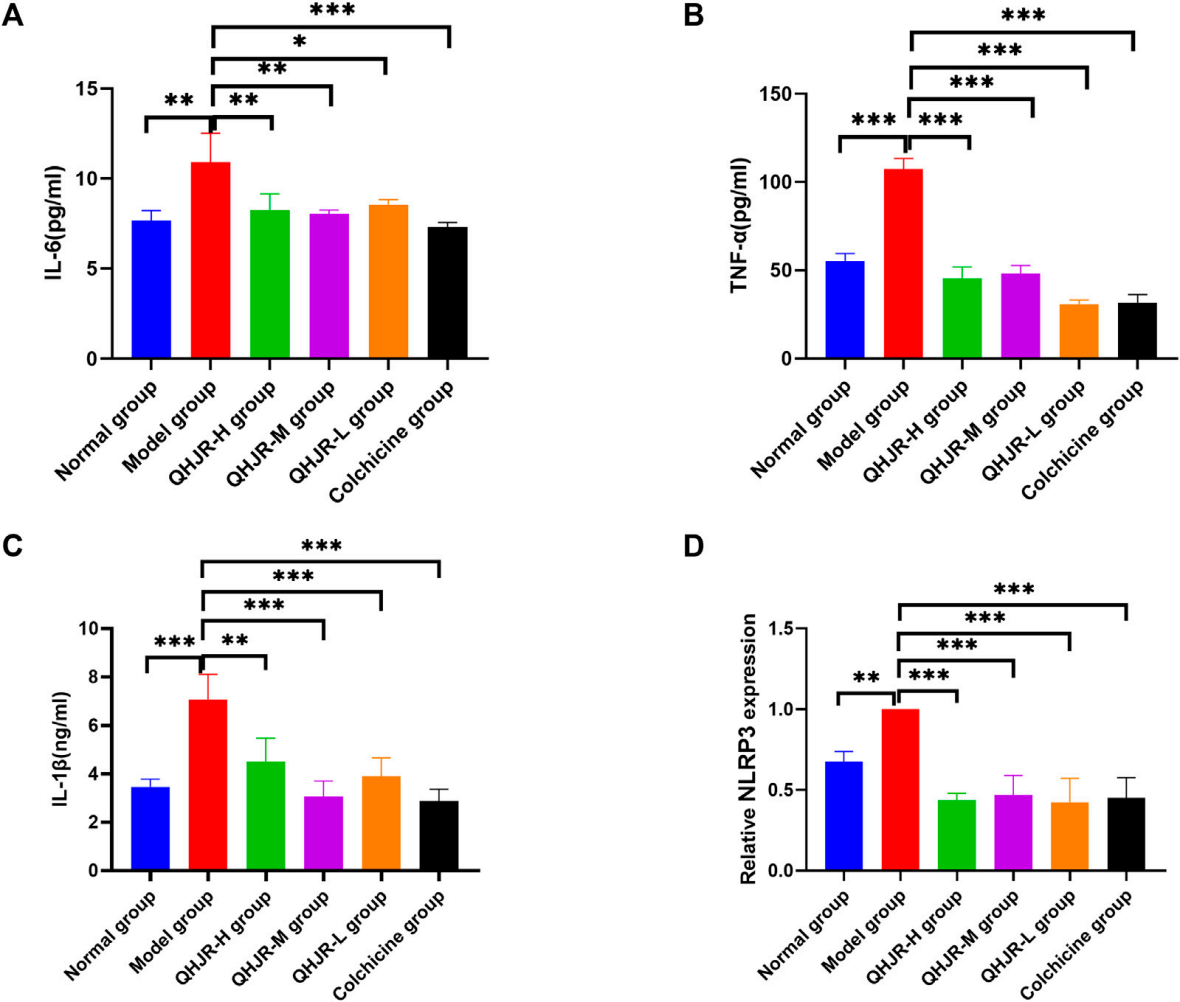
QHJR reduced the MSU-induced inflammation. **(A–C)** Inflammatory-related factor IL-6, TNF-α, and IL-1β levels through ELISA. **(D)** NLRP3 mRNA level was examined by qRT-PCR. (*p < 0.05, **p < 0.01, ***p < 0.001).

The results of the Biological function analysis suggested that AGA was closely related to inflammation, such as leukocyte chemotaxis, leukocyte migration, NOD-like receptor signaling pathway, and TNF signaling pathway. etc. Especially in the predicted hub genes of AGA, TNF, IL-6, and IL-1β were identified as joint targets of QHJR, suggesting that the primary mechanism underlying the treatment of QHJR for anti-AGA may reduce inflammation. To investigate this further, we analyzed the effects of QHJR on the level of inflammation markers TNF-α, IL-6, and IL-1β using the ELISA method. Our results demonstrate that QHJR treatment significantly inhibited the level of TNF-α, IL-6, and IL-1β induced by MSU (Figures 8A–C). In addition, qRT-PCR analysis revealed that NLRP3 levels significantly elevated depending on MSU-induced (Figure 8D). Compared with the Model group, QHJR treatment significantly downregulated the level, and its trend was consistent with colchicine (Figure 8D). These findings indicate that QHJR inhibits the inflammatory response and may represent an effective treatment for AGA. In summary, our study highlights the importance of targeting the inflammatory response as a key therapeutic approach for AGA.

### 3.8 QHJR anti-AGA by activating autophagy

**FIGURE 9:**
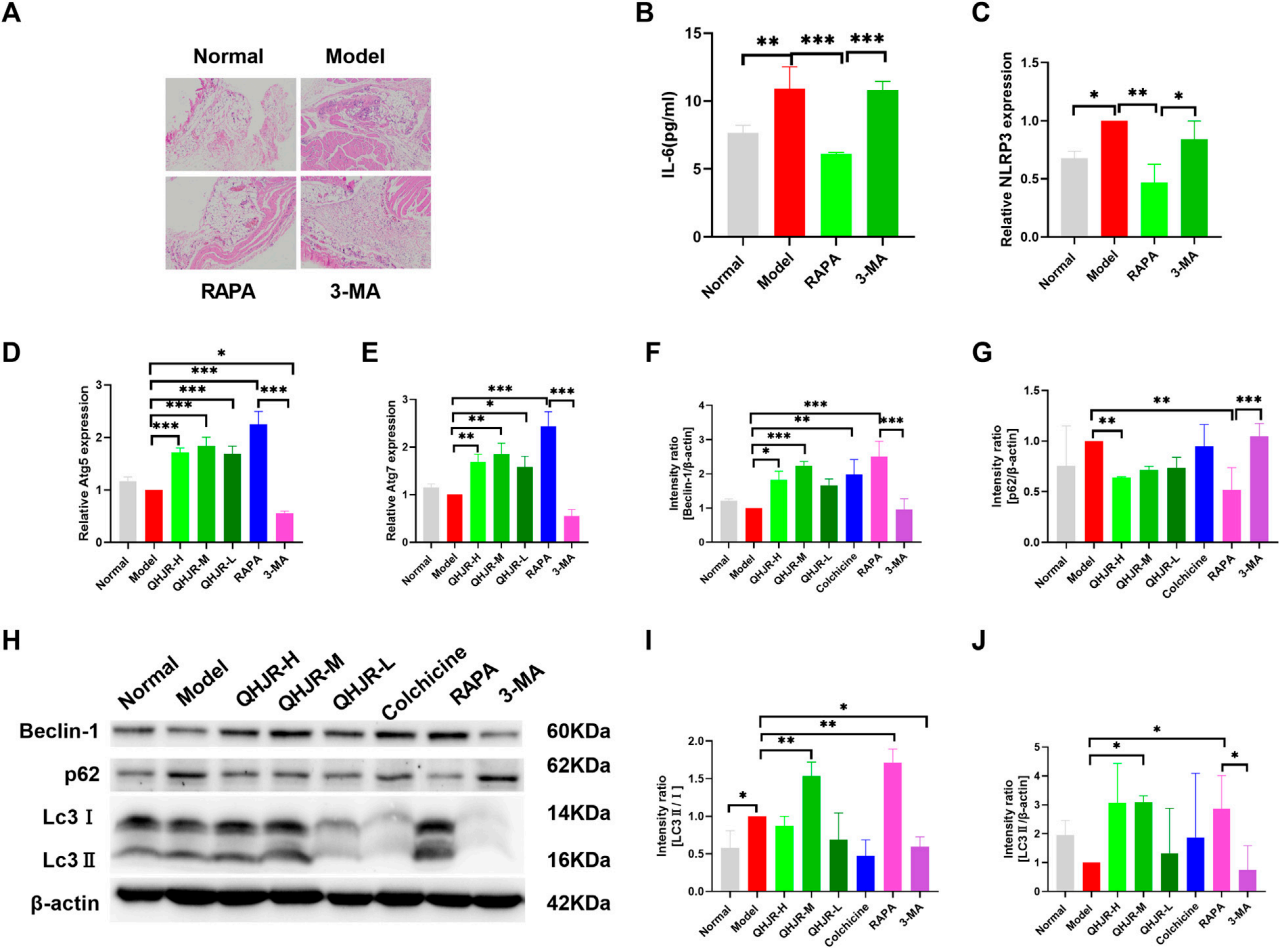
QHJR was involved in autophagy to regulate inflammation. **(A)** H&E method was adopted for detecting the expression of inflammation and autophagy. **(B)** Results of IL-6 expressions by Elisa. **(C–E)** The expressions of NLRP3, Atg5, and Atg7 were examined by RT-qPCR. **(F,H)** Results of Beclin-1/β-actin expressions by WB. **(G,H)** Results of p62/β-actin expressions by WB. **(I,J,H)** Results of LC3Ⅱ/Ⅰ and LC3Ⅱ/β-actin expressions by WB. (*p < 0.05, **p < 0.01, ***p < 0.001).

Autophagy has been reported to lower the inflammation. To investigate how autophagy influenced the treatment of AGA, autophagy level was first measured in inflammation. We added 3-MA which is an autophagy inhibitor, and RAPA which is an autophagy promoter as the controls. Many ATG proteins have vital effects on autophagy. Atg5 plays an essential part in canonical autophagy and autophagy-related processes (Masud et al., 2019). Interaction between p62/SQSTM1(p62) and light chain 3 (LC3) has an important impact on autophagosome generation and aggregated protein degradation in autophagosomes and lysosomes (Lyu et al., 2021). Compared with the Model group, the findings showed that Atg5 and Atg7 mRNA (Figures 9D,E) and LC3Ⅱ/Ⅰ protein (Figures 9H,I) levels dramatically increased in the RAPA group, and Atg5 mRNA (Figures 9D,E) and LC3Ⅱ/Ⅰ protein (Figures 9H,I) level significantly declined in 3-MA group. It was shown that the level of p62 was obviously inhibited in the RAPA group in comparison with the Model group and that the level of p62 was significantly enhanced in the 3-MA group relative to the RAPA group (Figures 9G,H). This was coincident with the role of 3-MA and RAPA.

Next, we examined the expression of autophagy in inflammatory-related factors. H&E assay displayed that the RAPA group had declined inflammatory cell infiltration relative to the Model group (Figure 9A). In addition, the RAPA group showed significantly declined inflammation relative to the Model group (Figure 9A). NLRP3 and IL-6 levels of the Model group were significantly increased relative to Normal group; IL-6 and NLRP3 levels were weakened in RAPA group compared with Model group (Figures 9B,C). Moreover, it was confirmed that promoting autophagy could reduce inflammation.

Moreover, the expressions of Atg5 and Atg7 (Figures 9D,E), Beclin-1, LC3Ⅱ/β-actin, and LC3Ⅱ/Ⅰ (Figures 9F,H–J) were increased in QHJR groups compared with Model group. The findings displayed that the level of p62 was lowered in QHJR groups compared with the Model group (Figures 9G,H). It suggested that QHJR might activate autophagy. We demonstrated that QHJR could reduce inflammation before, and QHJR activated autophagy, suggesting that QHJR could attenuate inflammation by activating autophagy.

### 3.9 QHJR activated autophagy via phosphorylating AMPK

**FIGURE 10:**
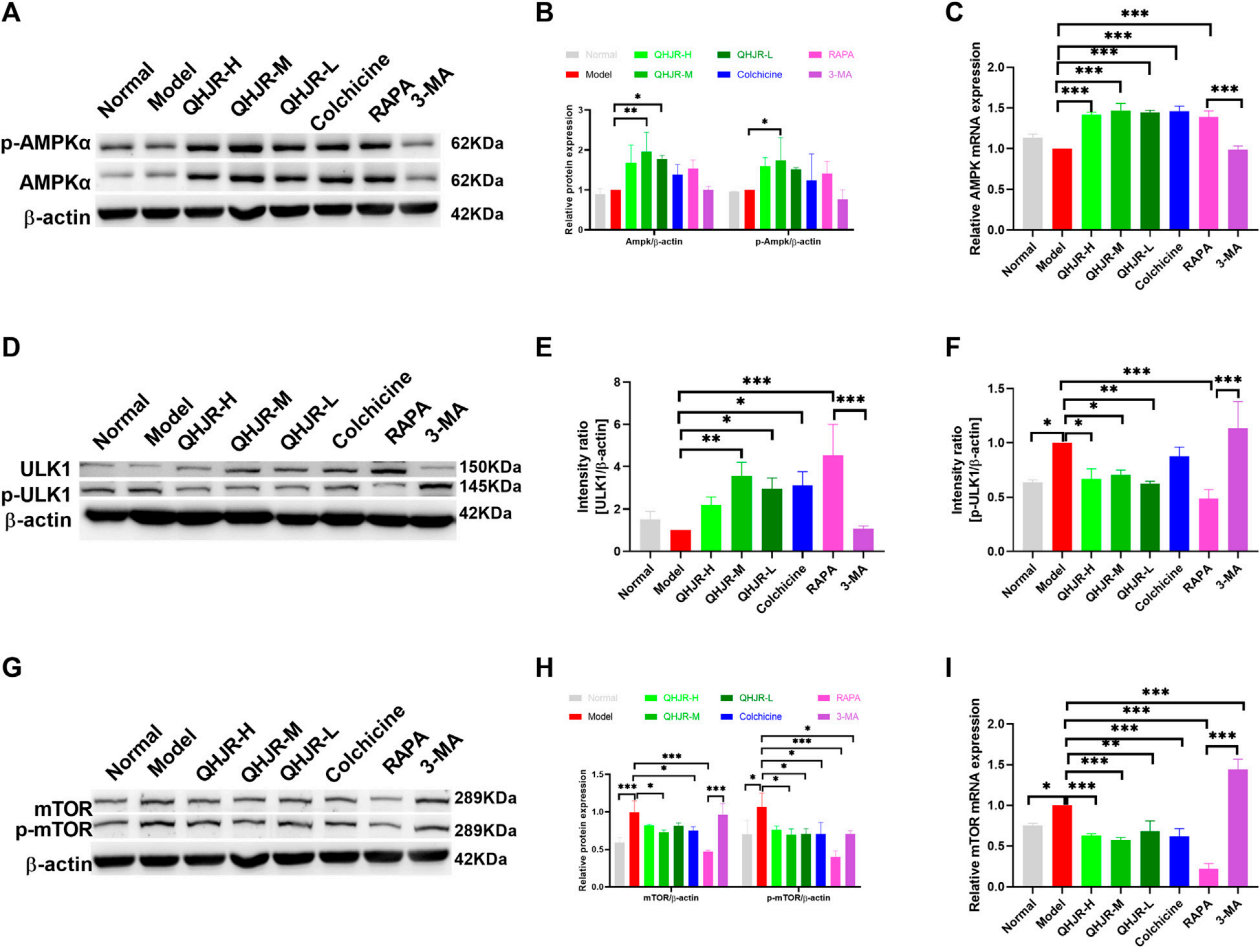
AMPK pathway was activated. **(A,B)** Results of AMPKα, p-AMPKα expression by WB. **(C)** Results of AMPK expression by qRT-PCR. **(D–F)** Results of ULK1, p-ULK1 expression by WB. **(G,H)** Results of mTOR, p-mTOR expression by WB. **(I)** Results of mTOR expression by qRT-PCR. (*p < 0.05, **p < 0.01, ***p < 0.001).

Considering that AMPKα is an energy sensor exerting a positive regulatory effect on autophagy by inhibiting mTOR to remove the phosphorylation inhibition of ULK1 at Ser757 and induce the binding of ULK1 to AMPK (Li and Chen, 2019), we aimed to confirm whether AMPK pathway has impacts on autophagy. WB and qRT-PCR assays were performed to examine AMPK-pathway-related levels in the RAPA and 3-MA groups. Compared with Model group, AMPKα and p-AMPKα1 (Thr 172) protein exhibited few significant changes in RAPA and 3-MA groups (Figures 10A,B), AMPK mRNA level (Figure 10C) was elevated in RAPA group, while levels of mTOR and p-mTOR (Ser2448) mRNA and protein (Figures 10F–H) and p-ULK1 (Ser 757) protein (Figures 10D–F) were descended in RAPA group; besides, ULK1 protein levels were elevated in RAPA group (Figures 10D–F). It displayed that the protein levels of mTOR, p-mTOR, and p-ULK1 (Figures 10D–I) and the mRNA level of mTOR (Figure 10H) were enhanced in the Model group compared with the Normal group. It suggested that AMPK might activate autophagy by suppressing p-ULK1 and p-mTOR.

AMPK phosphorylation at Thr172 exerts a vital role in activating AMPK, which usually acts as the activity indicator (Li and Chen, 2019). To verify whether AMPK was activated by QHJR, it was discovered that the p-AMPKα1 (Thr 172) protein level of the QHJR group increased relative to the Model group (Figures 10A,B). Compared with the Model group, it was shown that p-ULK1(Ser 757) and p-mTOR (Ser-2448) protein levels of the QHJR group decreased (Figures 10D,F–H). The activation of mTOR represents the well-recognized factor negatively regulating autophagy activation, and mTOR (Ser-2448) phosphorylation can serve as the activity marker. The obtained findings demonstrated that the AMPKα/mTOR/ULK1 pathway was activated after being subjected to the treatment of QHJR. All the findings demonstrated that QHJR could delay inflammation which was MSU-induced by enhancing autophagy using AMPKα/mTOR/ULK1 pathway.

## 4 Discussion

Acute gouty arthritis, is a common ailment, particularly among the middle-aged and elderly demographics (Grazio, 2012; Guo et al., 2023). The pathophysiology of this condition originates from the excessive build-up of uric acid, a metabolic byproduct of purine in the human system, and subsequent crystallization within the joints, triggering an acute arthritic response (Grazio, 2012; Guo et al., 2023). Ordinarily, hepatic and renal metabolism disposes of uric acid (Guo et al., 2023). However, dysfunction in its production or elimination causes its retention, facilitating the formation of urate crystal deposits, provoking inflammation, and pain, and significantly impacting the patient’s quality of life (Grazio, 2012). The disease’s onset may cause debilitating symptoms such as severe pain, joint dysfunction, redness, and fever (Grazio, 2012). The condition’s chronicity leads to soft tissue and joint injury, ultimately resulting in deformities and compromised function (Xu Y. T. et al., 2020). Additionally, gout triggers urate nephropathy, cardiovascular disease, metabolic syndrome, and other medical complications, further worsening the patient’s health (Li et al., 2019; Cohen-Rosenblum et al., 2022).

Currently, due to the increasing incidence rates and limitations of Western medicine (such as toxic side effects) (Nuki, 2008), the development of TCM has offered hope to patients. However, a limitation of TCM is that most CHM products do not possess up-to-date data regarding their safety and modern scientific evidence for their claimed clinical uses (Zhou J. et al., 2019). As a result, more research is needed to confirm the efficacy of TCM treatments for gouty arthritis. TCM has played a role in arthritis. For example, according to Lei Zhang et al. (Zhang W. et al., 2019), Total glucosides of paeony (TGP) and Paeoniflorin (Pae) generated immunomodulation and anti-inflammation by inhibiting the activation of synoviocytes and immunocytes, reducing inflammatory factor generation while reversing the aberrant signaling in synoviocytes. Menglin He et al. (He et al., 2022) considered Gentiopicroside (GPS) as treating AGA by reducing inflammation and pain. Xvwu Qian et al. (Qian et al., 2023) found that Atractylodes macrocephala played a good role in reducing hyperuricemia and inflammation. This is consistent with our study, as our research demonstrates that MSU-induced inflammation is upregulated in AGA, but expression levels decrease after QHJR treatment. Furthermore, the trend is consistent with the effects of colchicine treatment, suggesting the effectiveness of QHJR in AGA. To explore the specific mechanisms by which QHJR acts on AGA, we conducted further investigations.

Chinese herbal compounds exhibit remarkable therapeutic efficacy against various diseases. Their intricate composition, multifaceted therapeutic targets, and intricate treatment modalities present formidable challenges to the comprehensive elucidation of their underlying mechanisms. In recent times, network pharmacology has risen to prominence as a favored methodology for dissecting the intricate mechanisms of action inherent to complex Traditional Chinese Medicine (TCM) formulations (Wang et al., 2020b; Liu et al., 2023). OB and DL values are based on general rules and assumptions about compound properties, such as molecular weight, lipophilicity, and solubility. However, these values may not always accurately predict a compound’s behavior in the body or its potential as a drug (Zhu et al., 2013; Lage et al., 2018; Hoare, 2021). Chinese herbal compounds lacking proper pharmacokinetic properties would fail to reach their target organs and thereby hinder the expression of their biological effects (Mao et al., 2017; Liu et al., 2023). It has been empirically demonstrated that compounds with an oral bioavailability (OB) of ≥30% and a drug-likeness (DL) index ≥0.18 can be absorbed and distributed within the human body, thus earning the designation of pharmacokinetically active (Xu et al., 2012; Tao et al., 2013; Liu et al., 2023). The considerable therapeutic effects of QHJR on AGA within the compound-key targets network may be attributed to the presence of compounds with high degrees (Liu et al., 2023). Using network analysis, we initially predicted the metabolites in QHJR that could potentially target AGA. Subsequently, after performing chemical composition identification of QHJR using HPLC-Q-Orbitrap-MS, we found 27 metabolites, which revealed that 6 metabolites (quercetin, luteolin, formononetin, naringenin, taxifolin, diosgenin) were consistent with the predicted results which by Network Analysis. Additionally, we have identified five compounds (Neochlorogenic acid, Caffeic acid, Berberine, Isoliquiritigenin, and Formononetin) that, according to the TCMSP database, do not belong to any single herbal component of QHJR. Therefore, we infer that they result from interactions among the individual constituents of QHJR. Neochlorogenic acid has been studied for its potential effects on inflammation and is helpful in the treatment of rheumatoid arthritis (Gao et al., 2020). Caffeic acid, another caffeoylquinic acid derivative, has been studied for its effects on pain and inflammation in a murine model of gouty arthritis (Matosinhos et al., 2022). Recent studies have shown that berberine can inhibit the inflammatory response induced by monosodium urate (MSU) crystals and improve gouty arthritis symptoms (Liu et al., 2016). Berberine has also been shown to alleviate gouty arthritis in mice through the inhibition of xanthine oxidase activity (Xu et al., 2021). It was found that isoliquiritigenin inhibited rheumatoid arthritis, which is an inflammatory disease (Zhou and Wink, 2019). Another study reported that isoliquiritigenin is a potent inhibitor of NLRP3 inflammasome activation, which is implicated in the etiology of gouty arthritis (Lin et al., 2020). Formononetin has been shown to have significant effects on inflammation *in vitro* and animal models of many diseases, including rheumatoid arthritis and gouty arthritis (Zhang L. et al., 2019; Liu et al., 2022). TCMSP is still a valuable resource for drug discovery as it provides drug targets and diseases of each active compound, which can automatically establish the compound-target and target-disease networks that let users view and analyze the drug action mechanisms (Ru et al., 2014). However, the limitation of TCMSP is that it lacks some medicinal and pharmacological data, the dose-effect relationship of ingredients, and the drug diversity of components in each formula (Zhang W. et al., 2019). Thus, the generation of new metabolites by QHJR is merely a hypothesis, and further pharmacological experiments are required to validate this finding. Nevertheless, these metabolites do exist in QHJR, and numerous studies have confirmed that they possess varying degrees of decreasing inflammation, and have effects on AGA. This lays a foundation for the mechanism study of QHJR against AGA.

PharmMapper and SwissTarget, along with other target prediction databases, are essential tools for unraveling molecular interactions governing drug targets. These resources enable researchers to explore complex mechanisms and elucidate drug efficacy. With advanced algorithms and data integration, these platforms empower comprehensive investigation of the intricate drug-target relationship. Leveraging these databases, researchers gain insights into candidate drug therapeutics, driving novel pharmaceutical discovery. SwissTarget Prediction utilizes reverse screening to predict possible protein targets for drug candidates (Kanehisa et al., 2017; Wang et al., 2017). We conducted a Network analysis of the 27 metabolites identified through HPLC-Q-Orbitrap-MS. The results revealed that among these 27 metabolites, QHJR-10 (Caffeic acid), QHJR-16 (Isoliquiritigenin), QHJR-17 (cis-resveratrol), QHJR-7 (Ferulic acid), QHJR-8 (Apigenin), and QHJR-15 (Quercetin) exhibited the most prominent levels. We hypothesize that they may potentially be the key metabolites of QHJR in the treatment of AGA. Among them, QHJR-10 (Caffeic acid) and QHJR-16 (Isoliquiritigenin) mentioned earlier are the newly generated metabolites that may result from the interactions between the individual herbal components of QHJR. There is research that found that cis-resveratrol could reduce inflammation by inhibiting canonical and non-canonical inflammasomes in macrophages (Huang et al., 2014). Apigenin alleviates macrophage-polarization-induced inflammatory response (Ji et al., 2023). Apigenin has been shown to exert anti-inflammatory activity (Ouyang et al., 2021). Multiple studies have indeed demonstrated the effectiveness of ferulic acid (Doss et al., 2016; Ganesan and Rasool, 2019) and quercetin (Huang et al., 2012; Ruiz-Miyazawa et al., 2017) in treating AGA. However, due to their widespread distribution in the plant kingdom and their involvement in various disease processes, their therapeutic potential for multiple diseases has been extensively investigated through network analysis (Qi et al., 2020; Yang et al., 2020; Zhang et al., 2022). Further discussion on this topic will not be pursued extensively here. Furthermore, consistent with previous studies, these metabolites can alleviate AGA by reducing inflammation. However, computational models used in virtual screening may not always ensure that all predictions are correct (Sadybekov and Katritch, 2023). These key potential metabolites are currently only predictions, and their actual efficacy requires further experimental validation. This study aims to comprehensively investigate the mechanism of QHJR through an integrated approach of bioinformatic analysis and experimental validation.

The publicly available GEO platform was utilized to identify DEGs between healthy individuals and patients. The DEGs present promising therapeutic targets for combating the disease. Leveraging the GEO platform, researchers can gain critical insights into the molecular changes underlying the pathological condition, facilitating the development of targeted therapeutic interventions (Liao et al., 2018). Thus, the microarray data from GSE160170 in GEO were used to generate DEGs, forming the AGA target library. KEGG and GO tools were employed to analyze the molecular mechanisms and signaling pathways associated with QHJR drug therapies. This integrative approach aimed to unveil the complex molecular foundations and understand the therapeutic potential of QHJR (Kanehisa et al., 2017; Liu et al., 2018). Our findings indicated that the majority of enriched pathways with QHJR’s potential targets were associated with inflammation, suggesting that the suppression of inflammation might be a crucial aspect of QHJR’s treatment of AGA. Furthermore, through our comprehensive analysis of the PPI Network, we successfully identified key hub genes with high node degrees, namely, TNF, IL-1β, and IL-6. These hub genes not only hold significant relevance to inflammation but also emerged as the top candidates based on their centrality within the network. This finding further strengthens the hypothesis that these genes play crucial roles in the regulation and modulation of inflammatory processes. This is consistent with previous research. Previous studies have reported, Acute gouty arthritis is an inflammatory condition that is characterized by the deposition of monosodium urate (MSU) crystals in the joints (Busso and So, 2010; Zhang Q. B. et al., 2021). A study on human blood monocytes and synovial cells found that urate crystals stimulate the production of TNF-α, which is involved in the inflammatory process of gouty arthritis (Di Giovine, 1991). Amaral et al.found that TNF-α drives the expression of pro-IL-1β mRNA and IL-1β protein in experimental gout and that its transmembrane form is sufficient to trigger MSU-induced inflammation in mice (Amaral et al., 2016). Pro-inflammatory factors, esp. IL-1β; plays a vital role in gouty arthritis (Chang et al., 2017). When MSU crystals are absorbed by macrophages, they expedite IL-1β release. Recent clinical trials have shown that IL-1β blockade can reduce recurrent attacks of gouty arthritis (So et al., 2007; Dinarello, 2010). The upregulation of IL-1β, IL-6, and TNFα has been observed in patients with acute gouty arthritis (Busso and So, 2010; Zhang T. et al., 2021). NLRP3 inflammasome produces IL-1β in gout, composed of NLRP3, ASC, and pro-caspase-1 (Chang et al., 2017). W. Chang et al. (Wang et al., 2021) found that the expression of NLRP3 was upregulated in AGA. MicroRNA-223 has been found to suppress IL-1β and TNF-α production in gouty inflammation by targeting the NLRP3 inflammasome (Zhang T. et al., 2021). Our data displayed that inflammation level was significantly increased by the HE method. In addition, the level of NLRP3, TNF-α, IL-1β, and IL-6 was significantly increased after MSU induction, but QHJR and colchicine reversed this trend. This indicates that QHJR alleviates AGA by reducing inflammation. This is also consistent with the previous findings.

KEGG analysis indicates that apoptosis may be a key mechanism. Previous studies have shown that anti-inflammatory effects mediated by cell apoptosis are closely related to autophagy (Zhu and He, 2015), and a special factor called Beclin-1 is closely related to both cell apoptosis and autophagy (Zhu and He, 2015; Tang et al., 2022; Xu et al., 2022). Beclin-1 is the first mammalian autophagy gene (Xu et al., 2022). In addition to its involvement in autophagy, endocytosis, and phagocytosis (Zhu and He, 2015), Beclin-1 is also involved in the regulation of cell apoptosis and is expressed in various diseases, such as lung cancer (Zheng et al., 2020), colon cancer (Zhang et al., 2014), ovarian cancer (Cai et al., 2014), *etc.* Moreover, Beclin 1 is required for apoptotic cell engulfment and to coordinate actin dynamics and membrane phospholipid synthesis to promote efficient apoptotic cell engulfment (Konishi et al., 2012). Autophagy represents the self-defense mechanism in eukaryotes upon different survival pressures, and it is under precise modulation via different pathways, especially the AKT/mTOR pathway which is demonstrated to negatively regulate autophagy (Yang et al., 2021). Previous studies have reported that autophagy is closely related to GA which is MSU-induced (Piao et al., 2022). Altered ATG expression suggests that autophagy is involved in the pathogenesis of gouty arthritis (GA) and participates in regulating inflammation and metabolism (Dalbeth et al., 2019). However, conflicting evidence exists about the role of autophagy in GA. The autophagy-lysosomal pathway was found to be associated with gouty knee arthritis (Fu et al., 2023; Yuan et al., 2023). MSU crystal-induced acute gouty arthritis can be alleviated by autophagy induced by *p*P121 via inhibition of the NLRP3 inflammasome (Yuan et al., 2023). Autophagy contributes to the pathogenesis of gout through crosstalk with pyroptosis (Zhao L. et al., 2022). Uric acid suppresses autophagy and diminishes the anti-inflammatory capacity of the cell (Crisan et al., 2017). However, it is not specific whether autophagy expression is low or not in acute gouty arthritis. Our study displayed that p-mTOR and p-ULK1 were increased by AGA, suggesting that autophagy expression may be lowly expressed in AGA. This also confirms that autophagy is a fine regulatory mechanism. Autophagy plays a role in the regulation of inflammation and metabolism. Recent research has shown that autophagy negatively regulates proinflammatory responses and downregulates the production of cytokines such as IL-1β, IL-6, and TNF-α(Bussi et al., 2017). Autophagy modulates the transcription, processing, and secretion of IL-1β, acting as an important negative feedback mechanism for the regulation of inflammation (Harris, 2013). In our study, H&E staining analysis indicated that lymphoplasmacytic and other inflammatory levels were decreased by RAPA treatment. Inflammation-related factors (NLRP3, IL-6) confirmed that autophagy could decline inflammation which was MSU-induced. The findings suggested that autophagy could attenuate inflammation, consistent with previous studies. Our study suggested that autophagy-related factors (Atg5, Atg7, Beclin-1, LC3, p62) were activated after QHJR treatment, and QHJR upregulated autophagy expression. From this, we infer that QHJR may reduce inflammation and treat AGA by activating autophagy.

AMPK could regulate autophagy through phosphorylating autophagy-related factors, coordinately regulating ULK1 to induce autophagy with mTORC1 (Li and Chen, 2019). The previous studies demonstrated that medicine could treat arthritis by upregulating AMPK. Jing Zhou et al. (Zhou X. et al., 2019) found that BBR played the role of treating arthritis by upregulating the expression of phosphorylation AMPK. Yan Zhou et al. (Zhou et al., 2015) demonstrated that it suppressed SNP-mediated chondrocyte apoptosis while ameliorating cartilage degeneration by the activation of AMPK pathways and the inhibition of p38 MAPK activity. This study found that AMPKα and p-AMPKα1(Thr 172) had no obvious changes by RAPA and 3-MA treating, while p-ULK1 (Ser757) and p-mTOR (Ser2448) had obvious changes, confirming that AMPK might not directly adjust autophagy and AMPK might regulate autophagy through phosphorylating ULK1 and mTOR. This is slightly different from previous research. The data showed that the expressions of p-AMPKα1, p-ULK1, and p-mTOR expression were significantly activated after QHJR treatment. This suggests that QHJR may potentially alleviate inflammation and treat AGA by regulating the AMPKα/mTOR/ULK1 pathway to activate autophagy.

## 5 Conclusion

To sum up, all the findings confirmed that QHJR played an important role in AGA, and anti-inflammation is the main mechanism of QHJR for AGA. In our study, it was confirmed the high expression of the predicted key genes in AGA, and their expression was reduced after QHJR treatment, laying the foundation for the mechanism study of QHJR in the treatment of AGA. Following identification, we found the presence of 27 metabolites in QHJR, and the TCMSP database indicates that five of these metabolites do not belong to any single herbal component of QHJR. It is hypothesized that these metabolites may arise from interactions among the individual herbal components of QHJR, suggesting that QHJR cannot be substituted by any single herbal constituent. We found that AMPK may not directly activate autophagy. Furthermore, QHJR regulates autophagy by activating the AMPKα/mTOR/ULK1 pathway, thus reducing MSU-induced inflammation and contributing to the treatment of AGA. This provides novel insights and approaches for the clinical treatment of AGA.

However, this study still has limitations. For example, we found that there is no direct relationship between Ampk and autophagy, but further verification is needed. Furthermore, due to the limitations of network analysis, the predicted key potential metabolites still need further experimental validation for the accuracy of the research.

## Data Availability

The datasets presented in this study can be found in online repositories. The names of the repository/repositories and accession number(s) can be found below: https://www.ncbi.nlm.nih.gov/, 2020.

## Abbreviations

QHJR, Qingre-Huazhuo-Jiangsuan Recipe; RAPA, rapamycin; 3-MA, 3-Methyladenine; LC3, Light Chain 3; TCMSP, The TCM system pharmacology database; DEGs, differentially expressed genes; TCM, Traditional Chinese medicine; GO, Gene ontology; KEGG, Kyoto Encyclopedia of Genes and Genomes; GEO, Gene expression omnibus; PPI network, Protein-Protein Interaction network; HE, Hematoxylin-eosin staining assay; ELISA, Enzyme-linked immunosorbent assay; qRT-PCR, Quantitative Real-Time PCR; WB, Western blot.
